# Supplementary material for: Synthesis and Preliminary Characterization of Putative Anle138b-Centered PROTACs against α-Synuclein Aggregation
Source: Pharmaceutics. 2023 May 11;15(5):1467. doi: 10.3390/pharmaceutics15051467 (PMC10221781; doi:10.3390/pharmaceutics15051467)
Supplement: Supplementary file 1 [file pharmaceutics-15-01467-s001.zip › pharmaceutics-2352176-supplementary.pdf]

Synthesis and Preliminary Characterization of Putative Anle138b-Centered PROTACs against α-Synuclein Aggregation

Martina Pedrini [1,†], Angelo Iannielli [2,3,†], Lorenzo Meneghelli [1,‡], Daniele Passarella [1], Vania Broccoli [2,3,*] and Pierfausto Seneci [1,*]

[1] Chemistry Department, Milan University, Via Golgi 19, 20133 Milan, Italy; martina.pedrini@unimi.it (M.P.)
[2] Neuroscience Institute, Consiglio Nazionale delle Ricerche (CNR), Via Olgettina 58, 20132 Milan, Italy
[3] Stem Cells and Neurogenesis Unit, San Raffaele Hospital, Via Olgettina 58, 20132 Milan, Italy
* Correspondence: broccoli.vania@hsr.it (V.B.); pierfausto.seneci@unimi.it (P.S.); Tel.: +39-02-26434616 (V.B.); +39-02-50314060 (P.S.)
† These authors contributed equally to this work.
‡ Current address: BioCIS, Equipe de Chimie Biologique, CY Cergy Paris Université, 5 Mail Gay-Lussac, Neuville sur Oise, CEDEX,95031 Cergy, France.

SUPPORTING INFORMATION

Synthesis and analytical characterization of intermediates **1a-6**, and Anle138b-PROTAC constructs **7a,b, 8a,b** and **9a,b** *2-7*

NMR spectra of intermediates **1a-6**, and Anle138b-PROTAC constructs **7a,b, 8a,b** and **9a,b** *8-34*

Synthesis of Anle138b-based *m*-nitro **3a** and *p*-nitro-substituted diphenylpyrazole **3b**

The synthesis and analytical characterization of *m*-nitro derivative **3a** is described in the main text.

4′-Nitroacetophenone (1.00 g, 6.06 mmol, 2.0 eq.) was dissolved in dry toluene (4 mL) under N_2 atmosphere and cooled to 0 °C, under stirring. A 1M LiHMDS solution in THF (6.4 mL, 6.40 mmol, 2.1 eq.) was added quickly *via* syringe, and the resulting solution was stirred for 5 min. Then, acyl chloride (560 mg, 3.03 mmol, 1.0 eq.) dissolved in dry toluene (3.6 mL) was added dropwise to the stirred enolate solution at 0 °C. The reaction mixture was warmed to r.t and stirred for another 10 min, monitoring *via* TLC (eluent mixture: 6:4 n-hex/ EtOAc, developed in phosphomolybdic acid ethanolic solution). After reaction completion, AcOH (6.1 mL, 106 mmol, 35 eq.) was added in one portion, followed by EtOH (15.2 mL), THF (7.6 mL) and a 65% aqueous hydrazine solution (7.7 mL, 103 mmol, 34 eq.). The resulting solution was heated at reflux for 1 h, monitoring *via* TLC (eluent mixture: 6:4 n-hex/EtOAc, developed in phosphomolybdic acid ethanolic solution). After reaction completion and quenching with 1M NaOH (20 mL), the collected organic phases were extracted with EtOAc (70 mL), dried over Na_2SO_4, and concentrated *in vacuo*. The crude solid was purified *via* flash chromatography (eluent mixture: 7:3 n-hex/ EtOAc), affording pure compound **3b** (1.82 mmol, 65% yield) as a yellow solid.

Analytical characterization

3b:

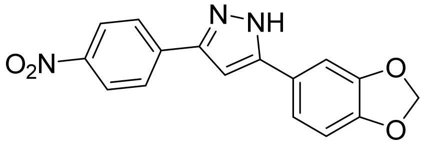

3b

^{1}H NMR (400 MHz, DMSO-d6): δ (ppm) = 13.56 (s, 1H), 8.29 (d, J = 8.2 Hz, 2H), 8.09 (d, J = 8.3 Hz, 2H), 7.39 (s, 1H), 7.37 – 7.29 (m, 2H), 7.03 (d, J = 8.0 Hz, 1H), 6.08 (s, 2H).

^{13}C NMR (101 MHz, DMSO-d6): δ (ppm) = 149.2, 147.9, 147.4, 146.4, 144, 143.8, 140.1, 125.8, 124.2, 119.1, 108.9, 105.7, 101.4, 100.6.

MS (ESI+), m/z calcd for $C_{16}H_{11}N3O_4$: 309.07, found 310.03 [M + H^+].

Synthesis of Anle138b-based *m*-anilino **1a** and *p*-anilino derivative **1b**

The synthesis and analytical characterization of *m*-anilino derivative **1a** is described in the main text.

p-Nitro-derivative **3b** (600 mg, 1.94 mmol, 1.0 eq.) was suspended in 5:1 EtOH/H_2O (19.4 mL) under stirring at r.t.. Fe powder (2.20 g, 38.8 mmol, 20.0 eq.) and a 6M H_2SO_4 aqueous solution (1.6 mL, 9.7 mmol, 5.0 eq.) were sequentially added to the reaction mixture, which was then heated and refluxed under stirring for 1 h. The reaction was monitored *via* TLC (eluent mixture: 4:6 n-hex/EtOAc, developed in phosphomolybdic acid ethanolic solution) and, after its completion, the hot solution was filtered over Celite and washed with hot EtOH (50 mL). Saturated aqueous $NaHCO_3$ (20 mL) was then added to quench the acid. The collected organic phases were extracted with EtOAc (50 mL), dried over Na_2SO_4, and concentrated *in vacuo*. The crude yellowish/orangish solid was purified *via* flash chromatography (eluent mixture: 4:6 n-hex/ EtOAc), affording pure target **1b** (1.20 mmol, 60% yield) as a light orange solid.

Analytical characterization

1b:

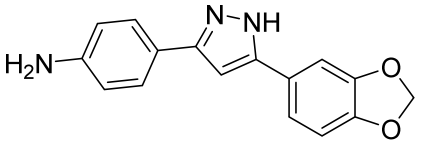

1b

^{1}H NMR (400 MHz, acetone-d6): δ (ppm) = 12.10 (s, 1H), 7.54 (d, *J* = 8.5 Hz, 2H), 7.40 – 7.33 (m, 2H), 6.89 (d, *J* = 8.4 Hz, 1H), 6.80 (s, 1H), 6.73 (d, *J* = 8.7 Hz, 2H), 6.02 (s, 2H), 4.82 (bs, 2H).
^{13}C NMR (101 MHz, acetone-d6): δ (ppm) = 150.2, 149.5, 149.1, 149.0, 148.3, 148.1, 128.6, 127.2, 119.9, 119.8, 115.3, 109.1, 106.6, 106.6, 102.0, 98.4.
MS (ESI^{+}), m/z calcd for $C_{16}H_{13}N_3O_2$: 279.10, found 280.25 [M + H^{+}].

Synthesis of Anle138b-based *m*-azido **2a** and *p*-azido derivative **2b**

The synthesis and analytical characterization of *m*-azido derivative **2a** is described in the main text.

p-Anilino derivative **1b** (176 mg, 0.63 mmol, 1.0 eq.) was suspended in 1:1 HCl/AcOH (1.3 mL) and cooled to 0 °C under stirring. A 1.8M $NaNO_2$ aqueous solution (1 mL, 1.89 mmol, 3.0 eq.) was added in one portion, and the reaction mixture was stirred at 0 °C for 15 min. Then, a 1.8M NaN_3 aqueous solution (1 mL, 1.89 mmol, 3.0 eq.) was slowly added under vigorous stirring. The reaction solution was warmed to r.t. and stirred for 1 h. The reaction was monitored *via* TLC (eluent mixture: 6:4 n-hex/EtOAc, developed in phosphomolybdic acid ethanolic solution) and, after its completion, was diluted with saturated aqueous $NaHCO_3$ (3 mL). The aqueous phase was extracted with EtOAc (4 x 10 mL), and the collected organic phases were dried over Na_2SO_4, and concentrated *in vacuo*. The crude orange solid was purified *via* flash chromatography (eluent mixture: 6:4 n-hex/AcOEt), affording pure *m*-azido target **2b** (0.42 mmol, 81% yield) as a brownish solid.

Analytical characterization
2b:

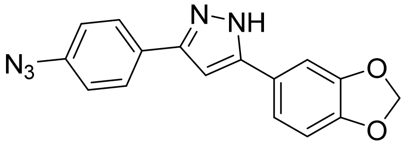

2b

^{1}H NMR (400 MHz, DMSO-d6): δ(ppm) = 13.22 (s, 1H), 7.85 (d, *J* = 8.1 Hz, 2H), 7.38 (s, 1H), 7.33 (d, *J* = 8.2 Hz, 1H), 7.19 (d, *J* = 8.1 Hz, 2H), 7.09 (s, 1H), 6.99 (d, *J* = 8.1 Hz, 1H), 6.06 (s, 2H).
^{13}C NMR (101 MHz, DMSO-d6): δ (ppm) = 157.7, 156.4, 152.3, 147.7, 127.4, 126.6, 119.5, 118.8, 108.6, 105.6, 101.1, 99.2.
MS (ESI^{+}), m/z calcd for $C_{16}H_{11}N_5O_2$: 305.09, found 306.16 [M + H^{+}].

Synthesis of lenalidomide alkynylamide linker-CRBN ligand construct **4**

The synthesis and analytical characterization of lenalidomide alkynylamide linker-CRBN ligand construct **4**is described in the main text.

Synthesis of PEGylated thalidomide alkynylamine linker-CRBN ligand construct **5**

Fluoro-thalidomide (151 mg, 0.55 mmol, 1.0 eq.) was dissolved under N_2 atmosphere in dry DMF (1.4 mL) under stirring at r.t.. PEGylated amine (90 µL, 0.60 mmol, 1.1 eq.) and DIPEA (190 µL, 1.09 mmol, 2.0 eq.) were dissolved in dry DMF (1.4 mL) under N_2 atmosphere and added to the

stirred solution. The reaction mixture was heated and stirred at 90°C for 8 h, monitoring *via* TLC (eluent mixture: 9:1 DCM/acetone, visualized under UV light). After its completion, the reaction mixture was quenched with H_2O (10 mL). The aqueous phase was extracted with EtOAc (4 x 10 mL), and the collected organic phases were dried over Na_2SO_4, and concentrated *in vacuo*. The crude black oil was purified *via* flash chromatography (eluent mixture: from 92:8 to 9:1 DCM/acetone), affording pure PEGylated thalidomide alkynylamine construct **5** (0.15 mmol, 28% yield) as a bright-yellow oil.

Analytical characterization

5:

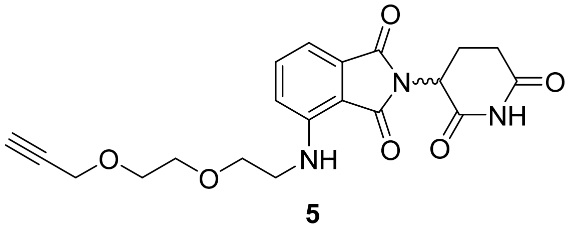

5

^{1}H NMR (400 MHz, acetone-d6): δ (ppm) = 9.90 (s, 1H), 7.56 (dd, *J* = 8.6, 7.0 Hz, 1H), 7.10 (d, *J* = 8.5 Hz, 1H), 7.03 (d, *J* = 7.1 Hz, 1H), 6.59 (t, *J* = 5.8 Hz, 1H), 5.07 (dd, *J* = 12.6, 5.4 Hz, 1H), 4.18 (d, *J* = 2.4 Hz, 2H), 3.73 (t, *J* = 5.4 Hz, 2H), 3.66 (s, 4H), 3.53 (q, *J* = 5.5 Hz, 2H), 3.02 – 2.86 (m, 3H), 2.85 – 2.70 (m, 2H), 2.26 – 2.16 (m, 1H).

Synthesis of thalidomide carboxylate linker-CRBN ligand construct **6**

Fluoro-thalidomide (106 mg, 0.38 mmol, 1.0 eq.) was dissolved under N_2 atmosphere in dry DMF (0.8 mL) at r.t.. 6-Aminohexanoic acid (60 mg, 0.46 mmol, 1.2 eq.) and DIPEA (140 µL, 0.77 mmol, 2 eq.) were dissolved in dry DMF (1 mL) and added to the stirred solution. The reaction mixture was then heated and stirred at 90 °C for 8 h, monitoring *via* TLC (eluent mixture: 9:1 DCM/MeOH, visualized under UV light). After its completion, the reaction was quenched with H_2O (10 mL). The aqueous phase was extracted with EtOAc (4x10 mL), and the collected organic phases were dried over Na_2SO_4, and concentrated *in vacuo*. The crude black oil was purified *via* flash chromatography (eluent mixture: from 97:3 to 95:5 DCM/MeOH), affording pure thalidomide carboxylate construct **6** (0.077 mmol, 20% yield) as a bright-yellow oil.

Analytical characterization

6:

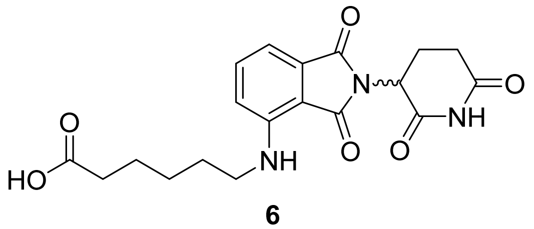

6

^{1}H NMR (400 MHz, acetone-d6): δ (ppm) = 10.49 (br, 1H), 9.88 (s, 1H), 7.58 (dd, *J* = 8.3, 7.3 Hz, 1H), 7.10 (d, *J* = 8.5 Hz, 1H), 7.02 (d, *J* = 7.1 Hz, 1H), 6.43 (t, *J* = 5.8 Hz, 1H), 5.06 (dd, *J* = 12.5, 5.4 Hz, 1H), 3.39 (td, *J* = 7.1, 5.8 Hz, 2H), 3.03 – 2.88 (m, 1H), 2.85 – 2.69 (m, 2H), 2.32 (t, *J* = 7.3 Hz, 2H), 2.24 – 2.15 (m, 1H), 1.78 – 1.59 (m, 4H), 1.55 – 1.42 (m, 2H).

Synthesis of Anle138b *m*/*p*-amide connected thalidomide PROTACs **7a,b**

m/*p*-Amino Anle138b **1a,b** (20 mg, 0.069 mmol, 1.0 eq.) and carboxylic acid **6** (40 mg, 0.103 mmol, 1.5 eq.) were dissolved under N_2 atmosphere in dry DMF (580 μL) under stirring at r.t.. HATU (30 mg, 0.069 mmol, 1.0 eq.) and DIPEA (40 μL, 0.206 mmol, 3 eq.) were sequentially added to the stirred solution. The reaction mixture was stirred at r.t. for 48 h and monitored *via* TLC (eluent mixture: 95:5 DCM/MeOH, visualized under UV light). After reaction completion, the reaction mixture was quenched with H_2O (5 mL), diluted with DCM (15 mL) and the collected organic phase was evaporated *in vacuo*. A crude yellow-green oil was purified *via* flash chromatography (eluent mixture: 97:3 DCM/MeOH), affording pure *m*-amide connected target **7a** (0.048 mmol, 70% yield) and pure *p*-amide connected target **7b** (0.026 mmol, 50% yield) as bright-yellow solids.

Analytical characterization

7a:

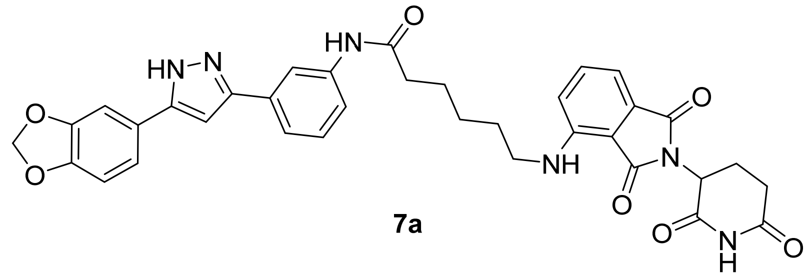

7a

^{1}H NMR (400 MHz, DMSO-d6): δ (ppm) = 13.17 (s, 1H), 11.09 (s, 1H), 9.96 (s, 1H8.06 (s, 1H), 7.61 – 7.50 (m, 2H), 7.46 (d, *J* = 7.7 Hz, 1H), 7.40 (d, *J* = 1.7 Hz, 1H), 7.37 – 7.31 (m, 2H), 7.10 (d, *J* = 8.6 Hz, 1H), 7.04 – 6.95 (m, 3H), 6.55 (t, *J* = 6.1 Hz, 1H), 6.06 (s, 2H), 5.04 (dd, *J* = 12.9, 5.3 Hz, 1H), 3.32 – 3.27 (m, 2H), 2.94 – 2.81 (m, 1H), 2.62 – 2.48 (m, 2H), 2.35 (t, *J* = 7.3 Hz, 1H), 2.06 – 1.96 (m, 1H), 1.72 – 1.56 (m, 4H), 1.47 – 1.34 (m, 2H).
^{13}C NMR (101 MHz, DMSO-d6**):** δ (ppm) = 172.9, 171.3, 170.2, 169.0, 167.3, 147.8, 147.0, 146.4, 139.7, 137.0, 135.7, 132.2, 118.3, 116.2, 111.5, 111.2, 110.4, 109.7, 109.4, 109.0, 106.2, 106.1, 105.2, 105.1, 101.2, 100.3, 99.9, 99.2, 98.5, 98.2, 49.1, 48.0, 41.8, 36.4, 28.6, 26.1, 24.9.
MS (ESI$^+$), m/z calcd for $C_{35}H_{32}N_6O_7$: 648.23, found 649.63 [M + H^+].

Analytical characterization

7b:

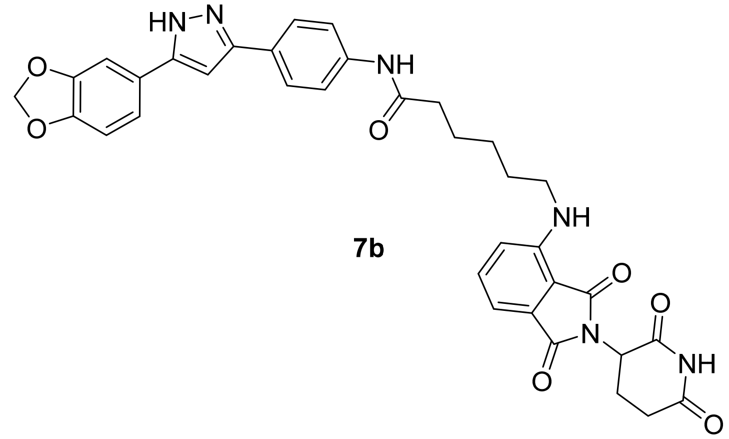

7b

^{1}H NMR (400 MHz, DMSO-d6): δ (ppm) = 11.09 (s, 1H), 9.96 (s, 1H), 7.72 (d, J = 8.6 Hz, 2H), 7.65 (d, J = 8.7 Hz, 2H), 7.57 (dd, J = 8.6, 7.1 Hz, 1H), 7.37 (d, J = 1.7 Hz, 1H), 7.32 (dd, J = 8.0, 1.7 Hz, 1H), 7.10 (d, J = 8.6 Hz, 1H), 7.06 – 6.94 (m, 3H), 6.54 (t, J = 5.9 Hz, 1H), 6.05 (s, 2H), 5.05 (dd, J = 12.9, 5.4 Hz, 1H), 3.32 – 3.27 (m, 2H), 2.96 – 2.81 (m, 1H), 2.63 – 2.49 (m, 1H), 2.35 (t, J = 7.5 Hz, 2H), 2.07 – 1.97 (m, 1H), 1.72 – 1.56 (m, 4H), 1.46 – 1.33 (m, 1H).
^{13}C NMR (101 MHz, DMSO-d6**):** δ (ppm) = 172.8, 171.2, 170.1, 169.0, 167.3, 147.7, 146.9, 146.4, 138.9, 136.3, 132.2, 125.4, 119.2, 118.8, 117.2, 110.4, 109.0, 108.6, 105.5, 101.1, 98.7, 53.4, 48.6, 41.7, 36.4, 31.0, 28.5, 26.0, 24.9, 22.2, 18.8, 12.6.

MS (ESI⁺), m/z calcd for $C_{35}H_{32}N_6O_7$: 648.23, found 649.63 [M + H⁺].

Synthesis of Anle138b *m*/*p*-triazole connected lenalidomide PROTACs **8a,b** and Anle *m*/*p*-triazole connected thalidomide PROTACs **9a,b**

The synthesis and analytical characterization of *m*-triazole connected lenalidomide PROTAC **8a** is described in the main text.

m/*p*-Azido Anle138b **2a,b** (50 mg, 0.16 mmol, 1.0 eq.) and alkynes **4** (70 mg, 0.20 mmol, 1.2 eq.) or **5** (80 mg, 0.20 mmol, 1.2 eq.) were dissolved in 1:1 DMF/H_2O (8 mL) under stirring at r.t.. After sequential addition of $CuSO_4{\cdot}5H_2O$ (8 mg, 0.03 mmol, 0.2 eq.) and Na-ascorbate (30 mg, 0.16 mmol, 1.0 eq.), the solution was stirred at r.t. for 5 h. The reaction was monitored *via* TLC (eluent mixture: 9:1 DCM/MeOH, developed in phosphomolybdic acid ethanolic solution), and after its completion was quenched with H_2O (10 mL). The aqueous phase was extracted with EtOAc (20 mL), and the collected organic phases were dried over Na_2SO_4, and concentrated *in vacuo*. The crude oil was purified *via* flash chromatography (eluent mixture: from 92:8 to 95:5 DCM/MeOH) or BIOTAGE® reverse phase chromatography (eluent: H_2O/ACN; from 0% ACN to 100% ACN), affording pure target PROTACs **8b** (0.023 mmol, 16% yield), **9a** (0.015 mmol, 20% yield) and **9b** (0.11 mmol, 87% yield) as bright yellow solids.

Analytical characterization

8b:

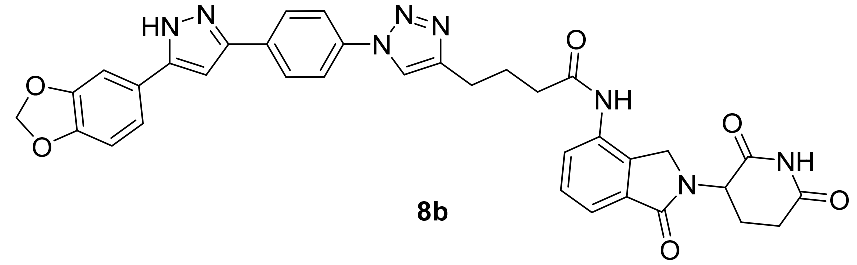

8b

¹H NMR (400 MHz, DMSO-d6): δ (ppm) = 11.01 (s, 1H), 9.82 (s, 1H), 8.67 (s, 1H), 8.02 (d, *J* = 8.4 Hz, 2H), 7.96 (d, *J* = 8.5 Hz, 2H), 7.84 (dd, *J* = 6.9, 2.1 Hz, 1H), 7.55 – 7.42 (m, 2H), 7.40 (d, *J* = 1.7 Hz, 1H), 7.35 (dd, *J* = 8.2, 1.7 Hz, 1H), 7.21 (s, 1H), 7.02 (d, *J* = 8.0 Hz, 1H), 6.07 (s, 2H), 5.14 (dd, *J* = 13.3, 5.1 Hz, 1H), 4.42 (d, *J* = 17.6 Hz, 1H), 4.35 (d, *J* = 17.5 Hz, 1H), 2.92 (ddd, *J* = 17.2, 13.6, 5.4 Hz, 1H), 2.80 (t, *J* = 7.4 Hz, 2H), 2.66 – 2.55 (m, 1H), 2.52 – 2.44 (under solvent peak, 2H**)**, 2.43 – 2.27 (m, 1H), 2.11 – 1.96 (m, 3H).
¹³C NMR (101 MHz, DMSO-d6**):** δ (ppm) = 172.9, 172.8, 167.8, 147.6, 133.8, 132.7, 128.6, 127.8, 126.3, 125.2, 121.4, 120.2, 120.1, 119.0, 118.9, 116.4, 109.7, 108.7, 105.6, 101.2, 99.7, 98.3, 82.8, 69.8, 69.8, 51.5, 46.5, 35.1, 31.2, 29.0, 24.7, 24.6, 22.6.
MS (ESI⁺), m/z calcd for $C_{35}H_{30}N_8O_6$: 658.23, found 659.65 [M + H⁺].

Analytical characterization

9a:

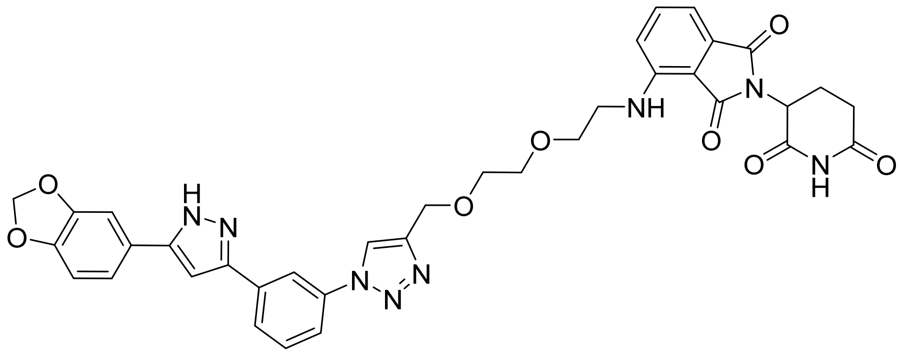

9a

^{1}H NMR (400 MHz, DMSO-d6): δ (ppm) = 11.07 (s, 1H), 8.85 (s,1H), 8.33 (s, 1H), 7.93 (d, J = 7.3 Hz, 1H), 7.83 (d, J = 7.9 Hz, 1H), 7.65 (t, J = 7.9 Hz, 1H), 7.55 (t, J = 8.3 Hz, 1H), 7.40 (d, J = 1.3 Hz, 1H), 7.35 (d, J = 8.2 Hz, 1H), 7.28 (s, 1H), 7.14 (d, J = 8.6 Hz, 1H), 7.06 – 6.98 (m, 2H), 6.61 (t, J = 5.3 Hz, 2H), 6.07 (s, 2H), 5.03 (dd, J = 12.9, 5.4 Hz, 1H), 4.67 – 4.61 (m, 2H), 3.69 – 3.60 (m, 6H), 3.51 – 3.44 (m, 2H), 2.89 – 2.79 (m, 1H), 2.62 – 2.48 (m, 2H), 2.04 – 1.96 (m, 1H).
^{13}C NMR (101 MHz, DMSO-d6**):** δ (ppm) = 172.8, 170.1, 168.9, 167.3, 162.9, 146.4, 145.2, 137.1, 136.2, 132.1, 130.4, 124.9, 118.9, 117.4, 116.5, 110.6, 108.7, 105.6, 101.2, 77.1, 72.5, 69.8, 69.7, 69.2, 68.9, 63.5, 48.5, 41.7, 40.2, 40.0, 39.8, 39.8, 39.7, 39.6, 39.6, 39.5, 39.4, 39.3, 39.2, 39.0, 30.9, 29.0, 27.9, 22.1.
MS (ESI^{+}), m/z calcd for $C_{36}H_{32}N_8O_8$: 704.23, found 705.72 [M + H^+].

Analytical characterization

9b:

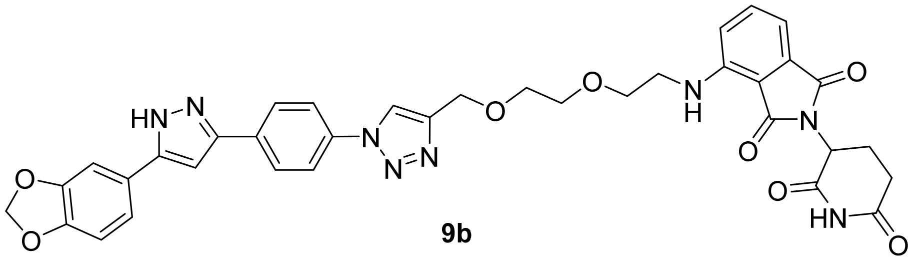

9b

^{1}H NMR (400 MHz, DMSO-d6): δ (ppm) = 13.31 (s, 1H), 11.08 (s, 1H), 8.81 (s, 1H), 8.05 – 7.93 (m, 4H), 7.56 (dd, J = 8.6, 7.1 Hz, 1H), 7.40 (d, J = 1.7 Hz, 1H), 7.21 (s, 1H), 7.14 (d, J = 8.6 Hz, 1H), 7.01 (d, J = 7.0 Hz, 2H), 6.62 (t, J = 5.8 Hz, 1H), 6.07 (s, 2H), 5.04 (dd, J = 12.9, 5.4 Hz, 1H), 4.64 (s, 2H), 3.65 (m, 6H), 3.53 – 3.44 (m, 2H), 2.86 (m, 1H), 2.62 – 2.51 (m, 4H), 2.01 (m, 1H).
^{13}C NMR (101 MHz, DMSO): δ (ppm) = 172.8, 170.1, 168.9, 167.3, 147.8, 146.4, 145.2, 136.2, 135.7, 132.1, 126.2, 122.0, 120.3, 118.9, 117.4, 110.6, 109.2, 108.7, 105.6, 101.2, 99.6, 69.7, 69.2, 68.9, 63.5, 48.5, 41.7, 31.0, 22.1.
MS (ESI^{+}), m/z calcd for $C_{36}H_{32}N_8O_8$: 704.23, found 705.65 [M + H^+].

Figure S1. ^{1}H and ^{13}C-NMR spectra of **3a**

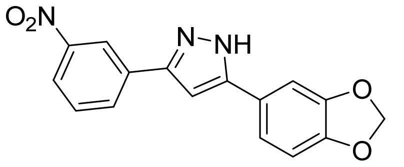

3a

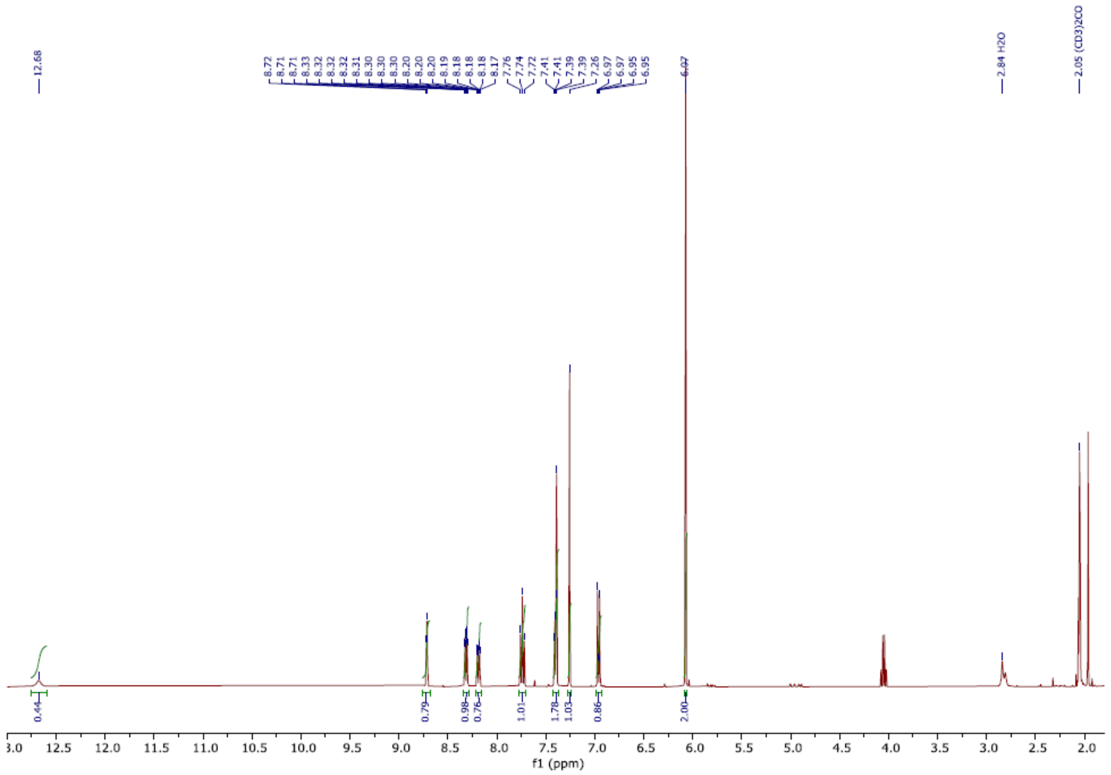

^{1}H NMR (400 MHz, acetone-d6): δ (ppm) = 12.68 (bs, 1H), 8.71 (t, *J* = 2.0 Hz, 1H), 8.31 (ddd, *J* = 7.8, 1.7, 1.0 Hz, 1H), 8.19 (ddd, *J* = 8.2, 2.3, 1.0 Hz, 1H), 7.74 (t, *J* = 8.0 Hz, 1H), 7.43 – 7.36 (m, 2H), 7.26 (s, 1H), 6.96 (d, *J* = 8.8 Hz, 1H), 6.07 (s, 2H).

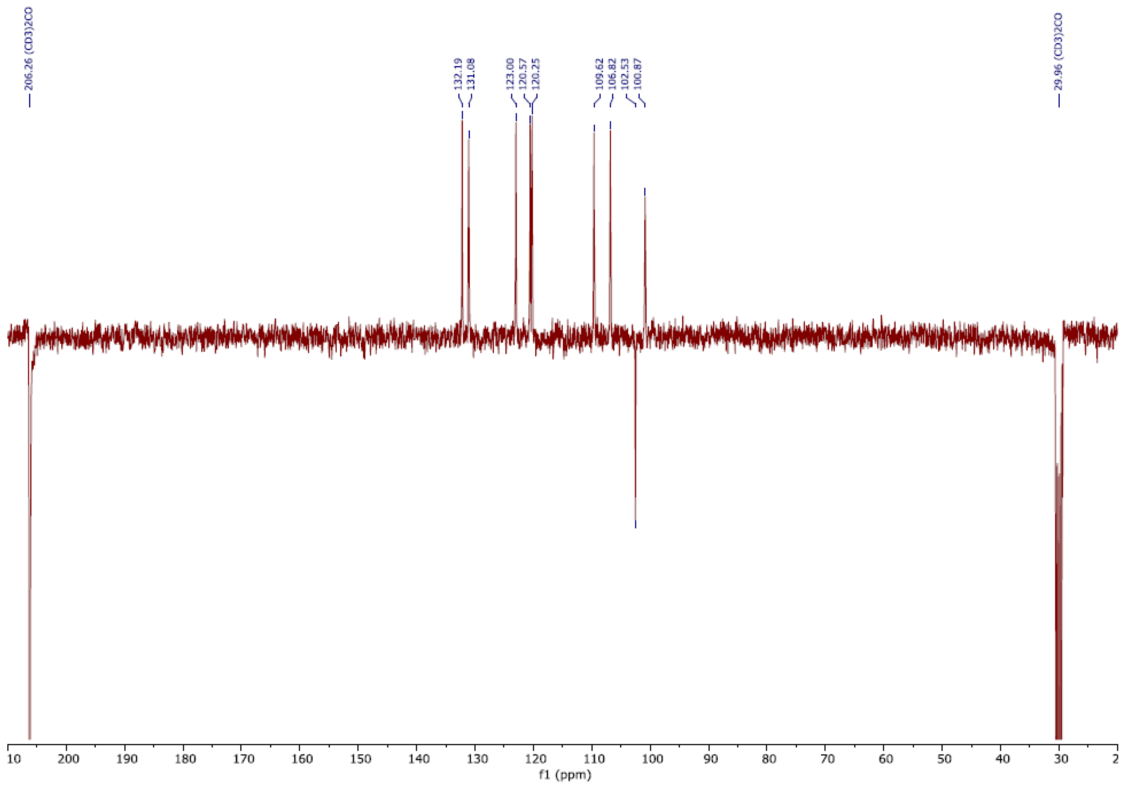

^{13}C NMR (101 MHz, acetone-d6): δ (ppm) = 132.2, 131.1, 123.0, 120.6, 120.3, 109.6, 106.8, 102.5, 100.9. Coherent with published data.[1]

Figure S2. ^{1}H and ^{13}C-NMR spectra of **3b**

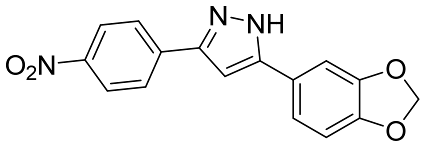

3b

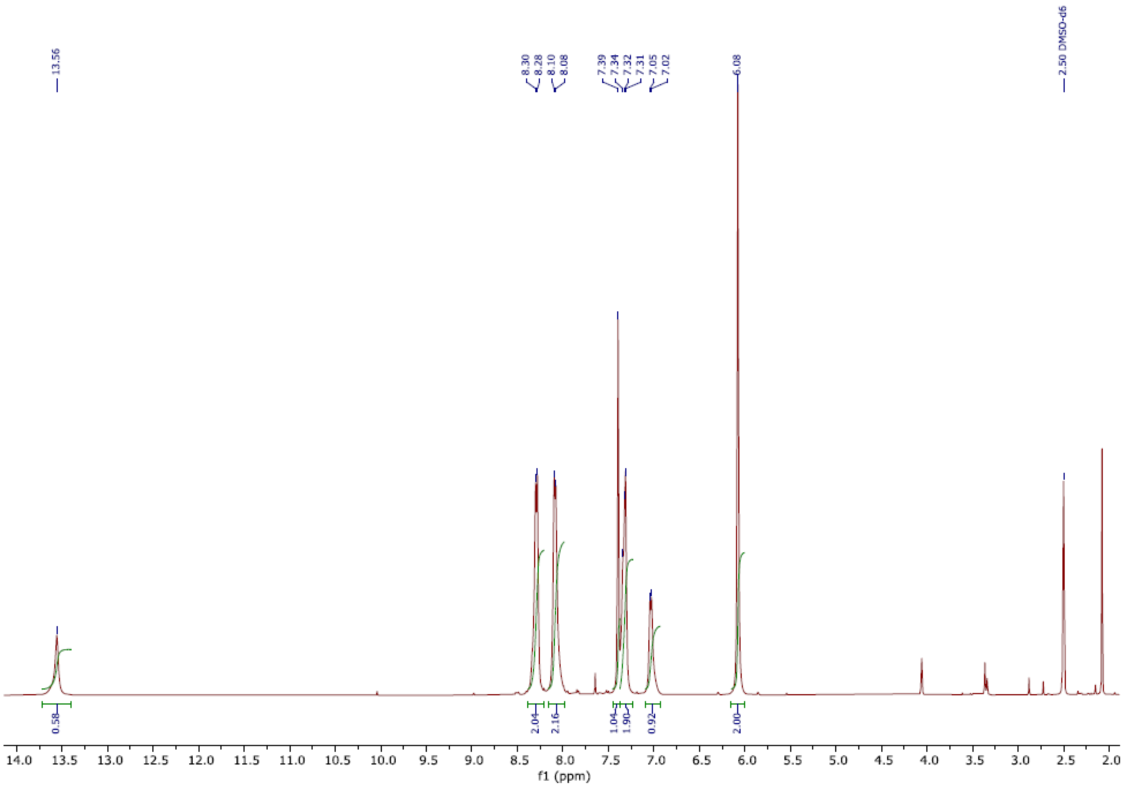

^{1}H NMR (400 MHz, DMSO-d6): δ (ppm) = 13.56 (s, 1H), 8.29 (d, J = 8.2 Hz, 2H), 8.09 (d, J = 8.3 Hz, 2H), 7.39 (s, 1H), 7.37 – 7.29 (m, 2H), 7.03 (d, J = 8.0 Hz, 1H), 6.08 (s, 2H).

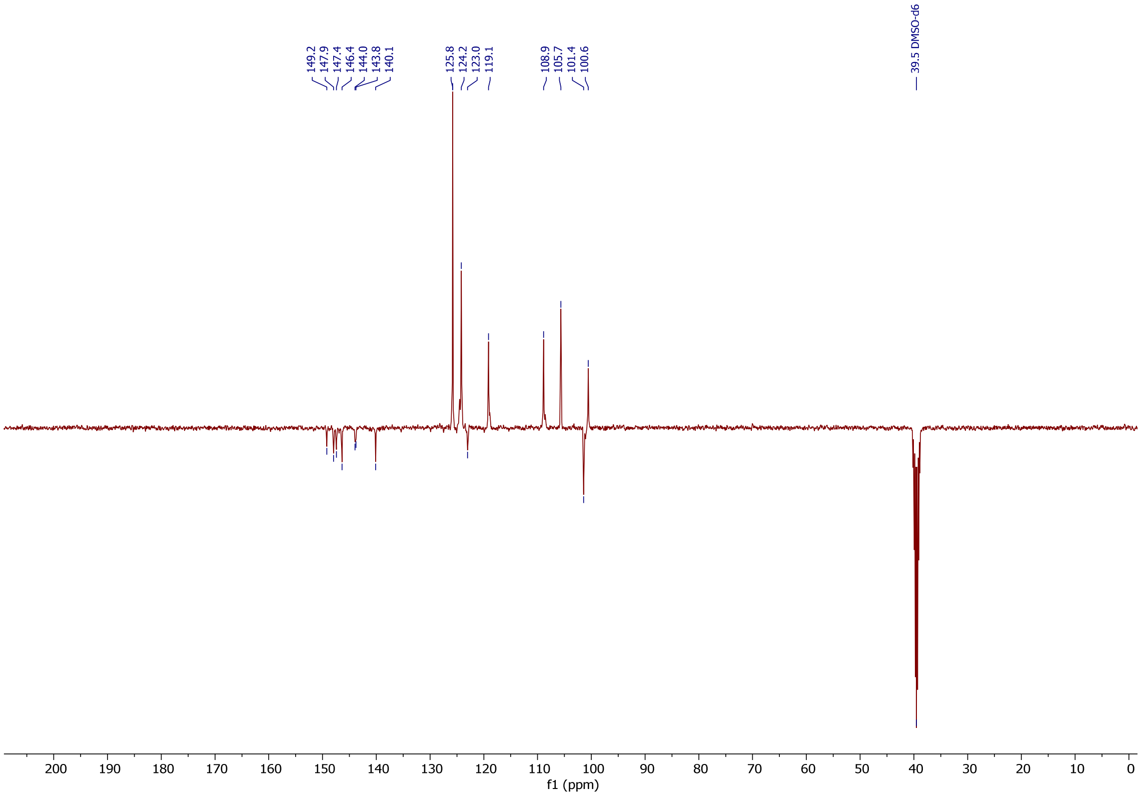

^{13}C NMR (101 MHz, DMSO-d6): δ (ppm) = 149.2, 147.9, 147.4, 146.4, 144, 143.8, 140.1, 125.8, 124.2, 119.1, 108.9, 105.7, 101.4, 100.6.

Figure S3. ^{1}H and ^{13}C-NMR spectra of **1a**

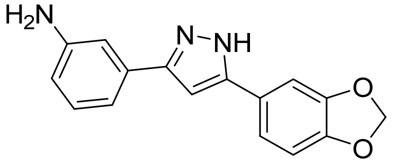

1a

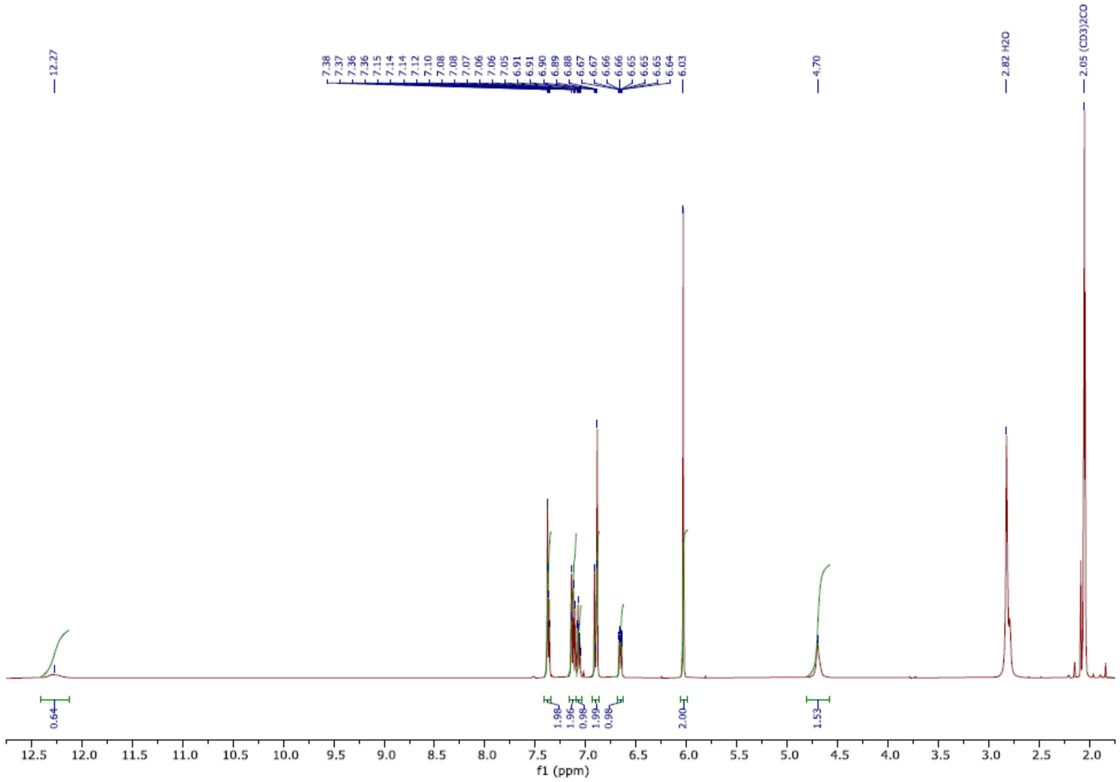

^{1}H NMR (400 MHz, acetone-d6): δ (ppm) = 12.27 (s, 1H), 7.41 – 7.33 (m, 2H), 7.14 (t, *J* = 1.5 Hz, 1H), 7.12 (t, *J* = 7.7 Hz, 1H), 7.07 (dt, *J* = 7.6, 1.4 Hz, 1H), 6.90 (d, *J* = 8.6 Hz, 1H), 6.88 (s, 1H), 6.66 (ddd, *J* = 7.8, 2.3, 1.2 Hz, 1H), 6.03 (s, 2H), 4.70 (s, 2H).

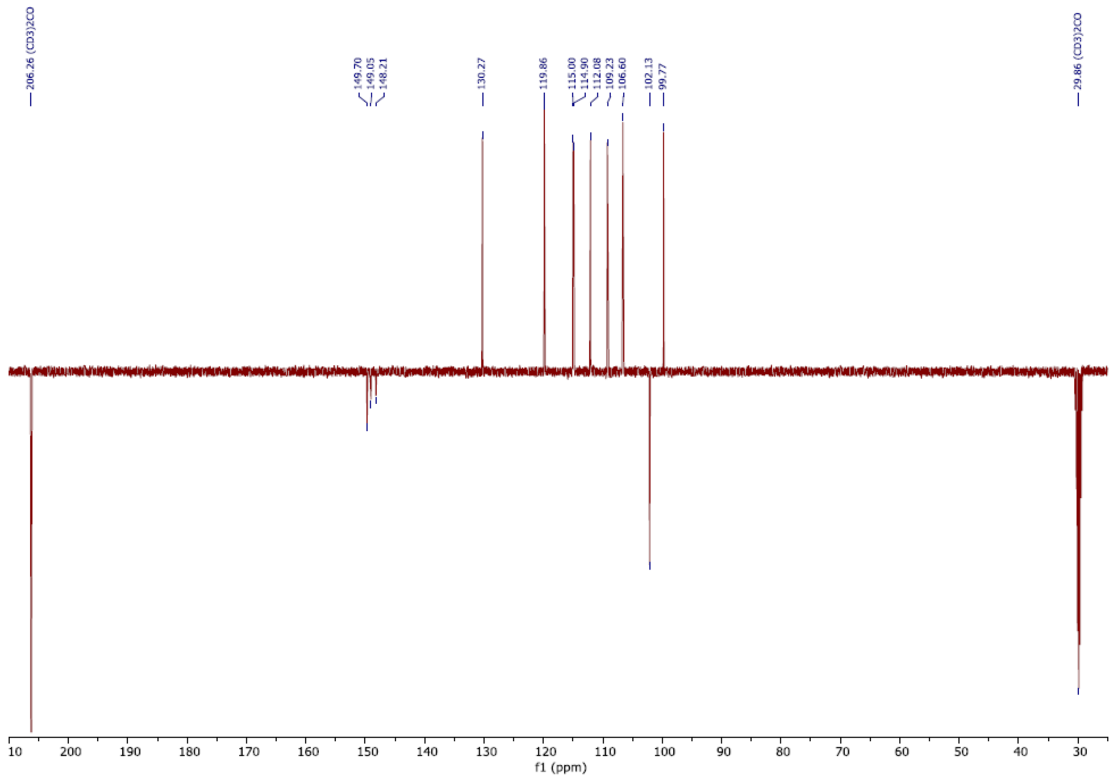

^{13}C NMR (101 MHz, acetone-d6): δ (ppm) = 149.7, 149.0, 148.2, 130.3, 119.9, 115.0, 114.9, 112.1, 109.2, 106.6, 102.1, 99.8. Coherent with published data.[1]

Figure S4. ^{1}H and ^{13}C-NMR spectra of **1b**

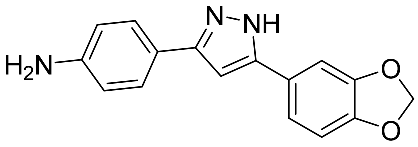

1b

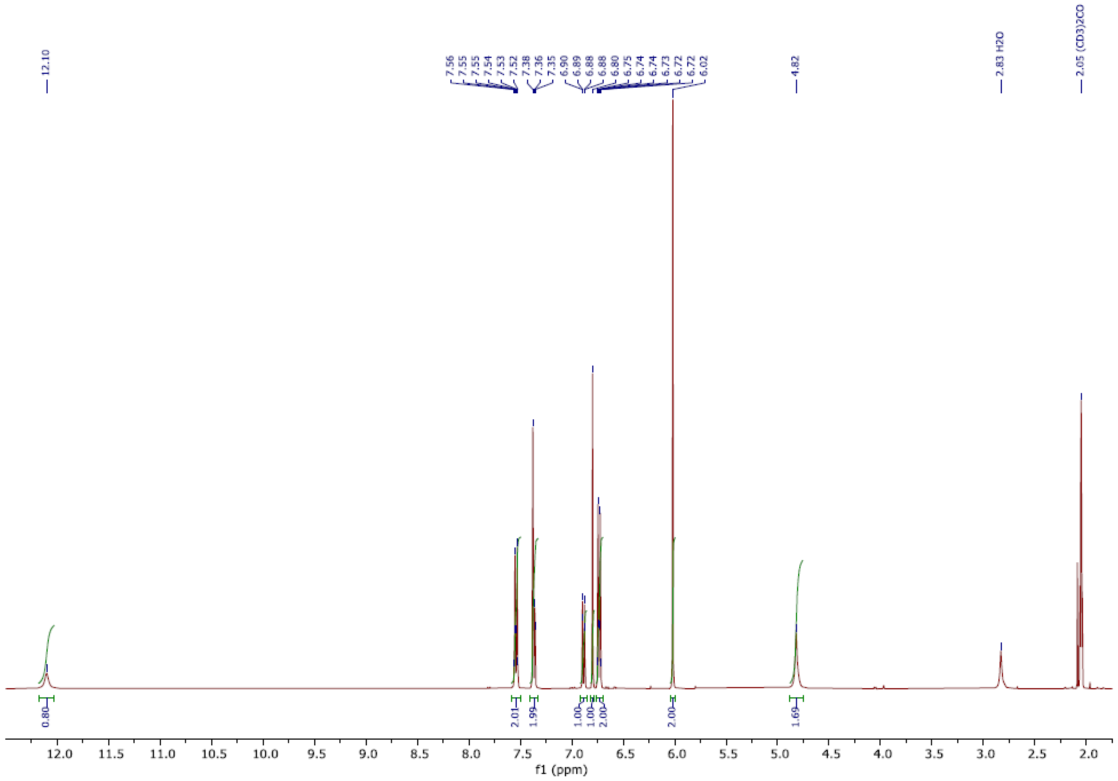

^{1}**H NMR** (400 MHz, acetone-d6): δ (ppm) = 12.10 (s, 1H), 7.54 (d, *J* = 8.5 Hz, 2H), 7.40 – 7.33 (m, 2H), 6.89 (d, *J* = 8.4 Hz, 1H), 6.80 (s, 1H), 6.73 (d, *J* = 8.7 Hz, 2H), 6.02 (s, 2H), 4.82 (bs, 2H).

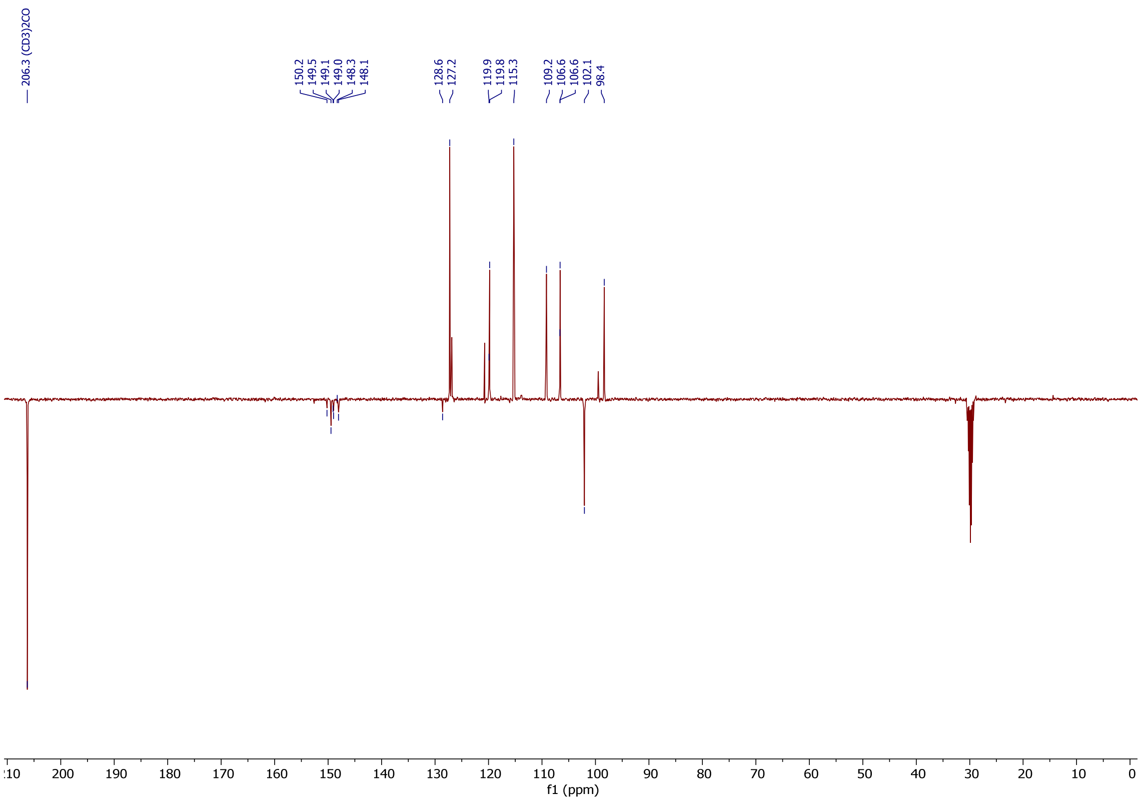

¹³C NMR (101 MHz, acetone-d6): δ (ppm) = 150.2, 149.5, 149.1, 149.0, 148.3, 148.1, 128.6, 127.2, 119.9, 119.8, 115.3, 109.1, 106.6, 106.6, 102.0, 98.4.

Figure S5. ^{1}H and ^{13}C-NMR spectra of **2a**

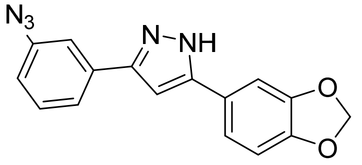

2a

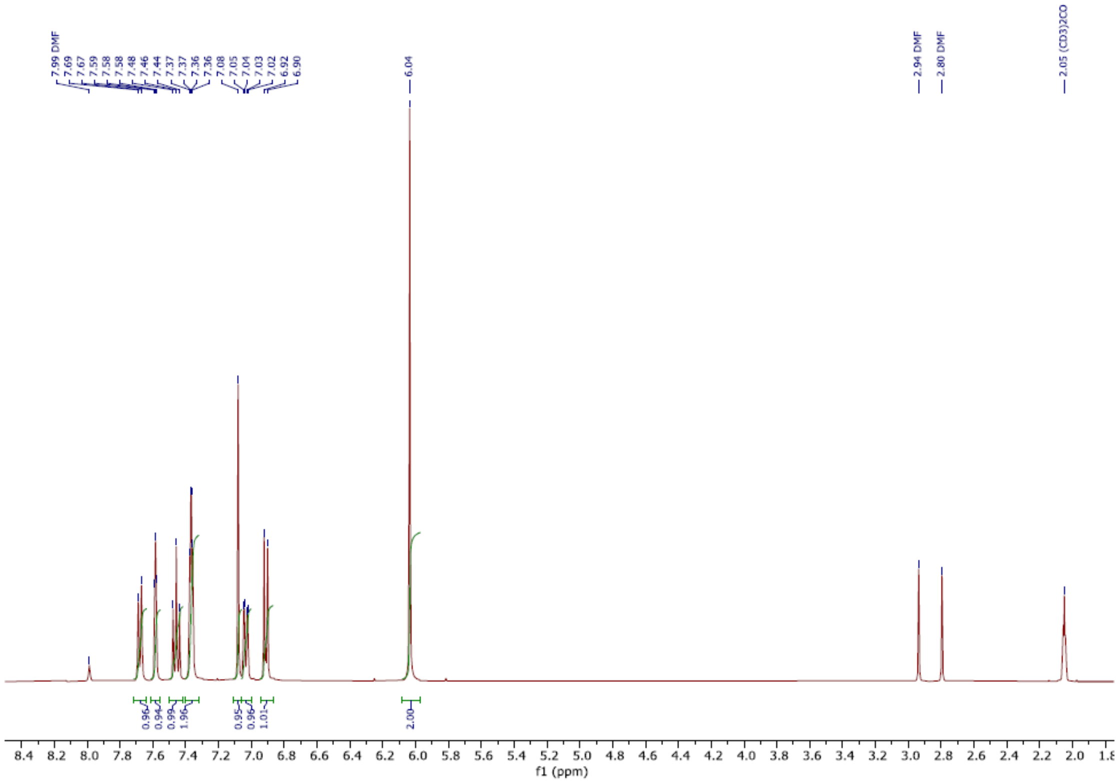

^{1}**H NMR** (400 MHz, acetone-d6): δ (ppm) = 7.68 (d, *J* = 7.8 Hz, 1H), 7.58 (t, *J* = 1.9 Hz, 1H), 7.46 (t, *J* = 7.9 Hz, 1H), 7.40 – 7.33 (m, 2H), 7.08 (s, 1H), 7.03 (dd, *J* = 7.9, 2.4 Hz, 1H), 6.91 (d, *J* = 8.5 Hz, 1H), 6.04 (s, 2H).

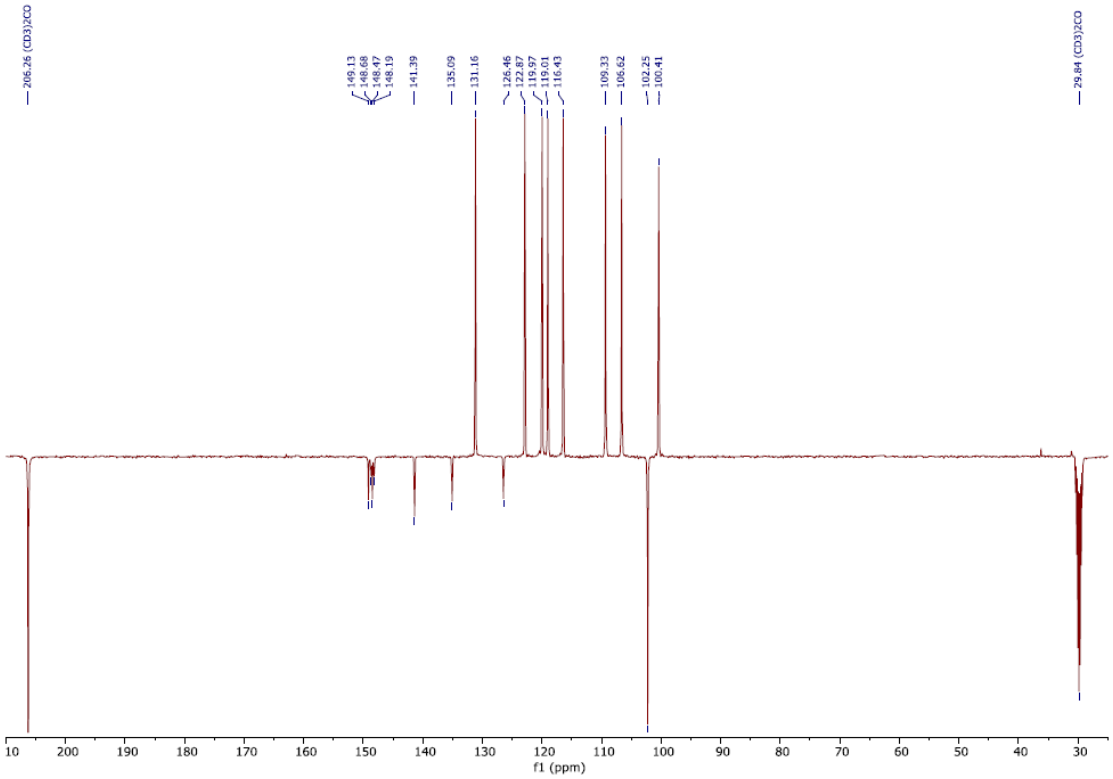

^{13}C NMR (101 MHz, acetone-d6): δ (ppm) = 149.1, 148.7, 148.5, 148.2, 141.4, 135.1, 131.2, 126.5, 122.9, 120.0, 119.0, 116.4, 109.3, 106.6, 102.2, 100.4.

Figure S6. ^{1}H and ^{13}C-NMR spectra of **2b**

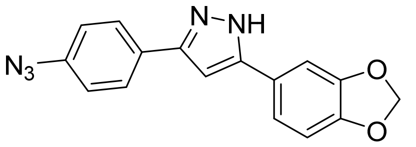

2b

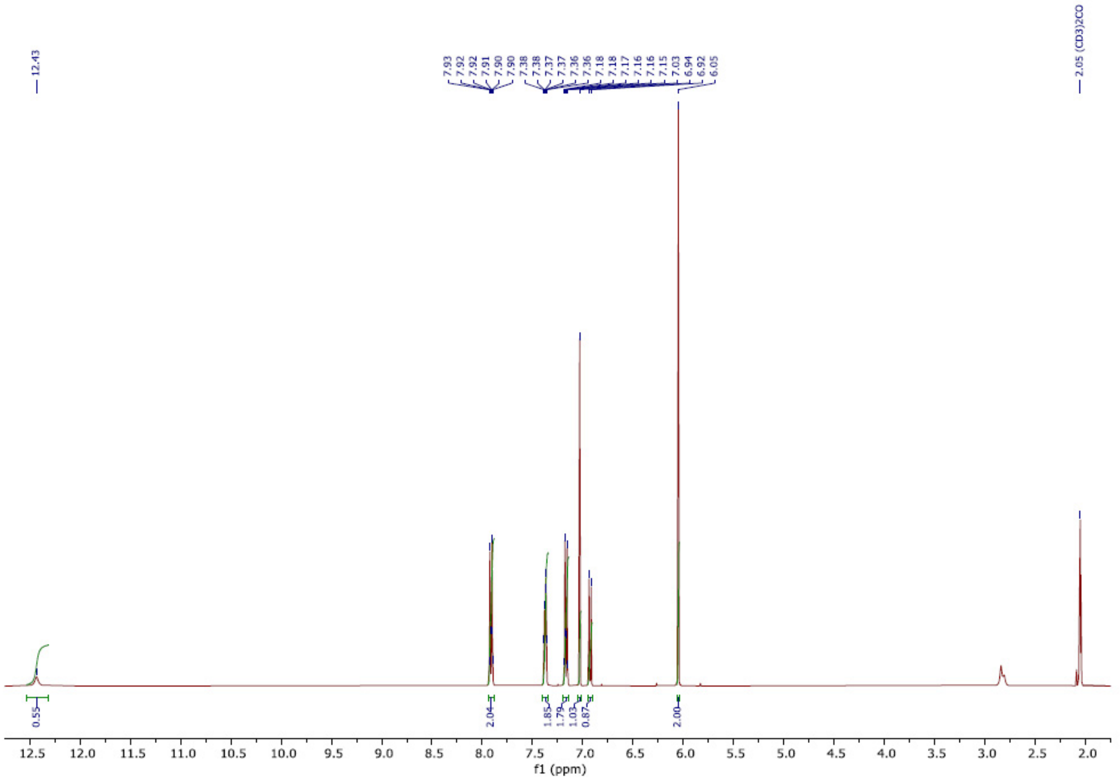

^{1}H NMR (400 MHz, DMSO-d6): δ(ppm) = 13.22 (s, 1H), 7.85 (d, *J* = 8.1 Hz, 2H), 7.38 (s, 1H), 7.33 (d, *J* = 8.2 Hz, 1H), 7.19 (d, *J* = 8.1 Hz, 2H), 7.09 (s, 1H), 6.99 (d, *J* = 8.1 Hz, 1H), 6.06 (s, 2H).

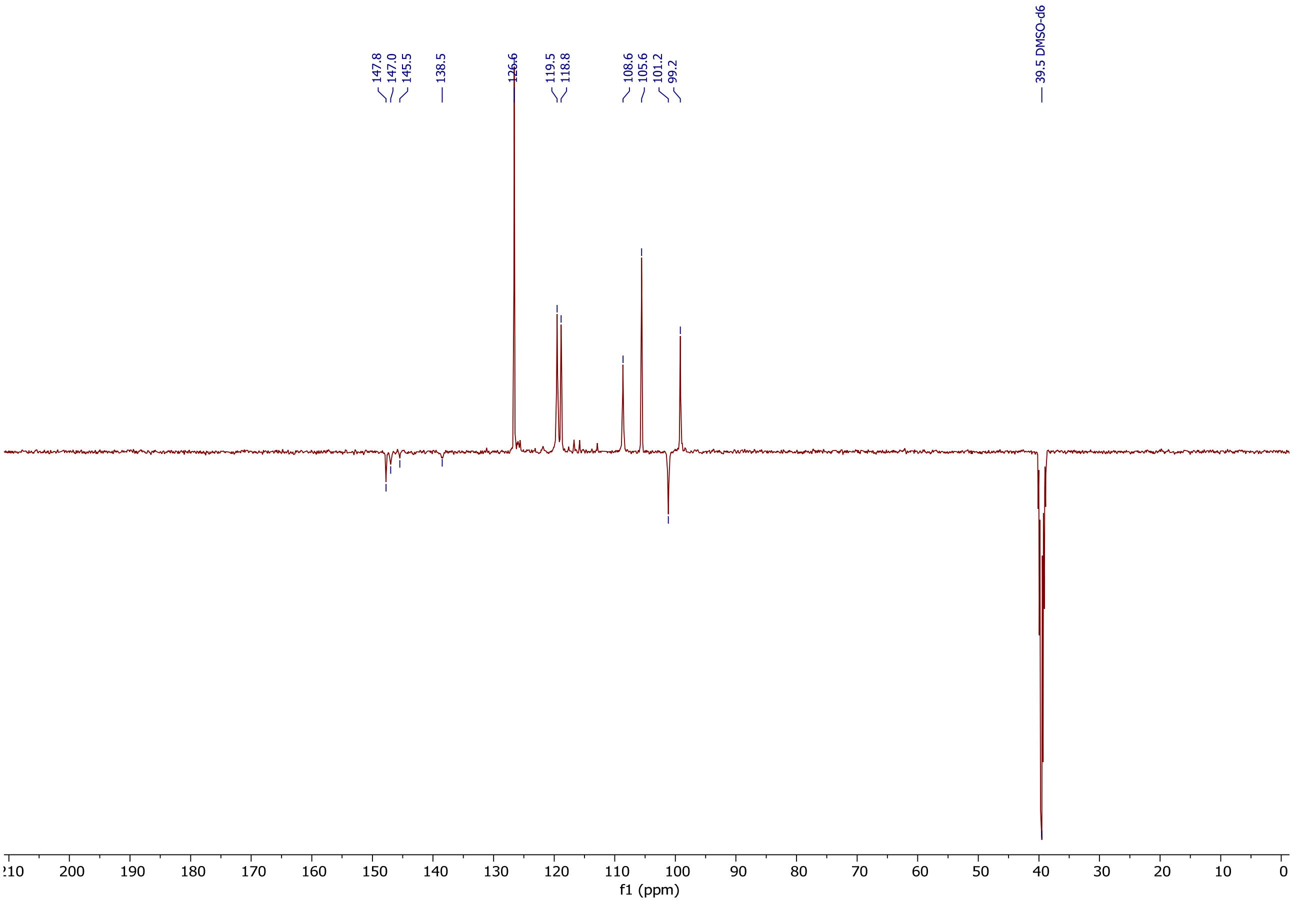

^{13}C NMR (101 MHz, DMSO-d6): δ (ppm) = 147.8, 147.0, 145.5, 138.5, 126.6, 119.5, 118.8, 108.6, 105.6, 101.2, 99.2.

Figure S7. ^{1}H-NMR spectra of **4**

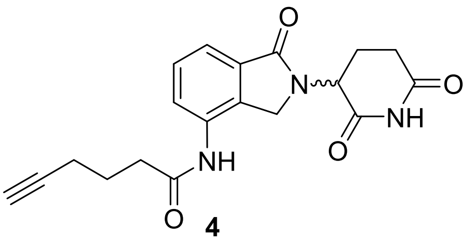

4

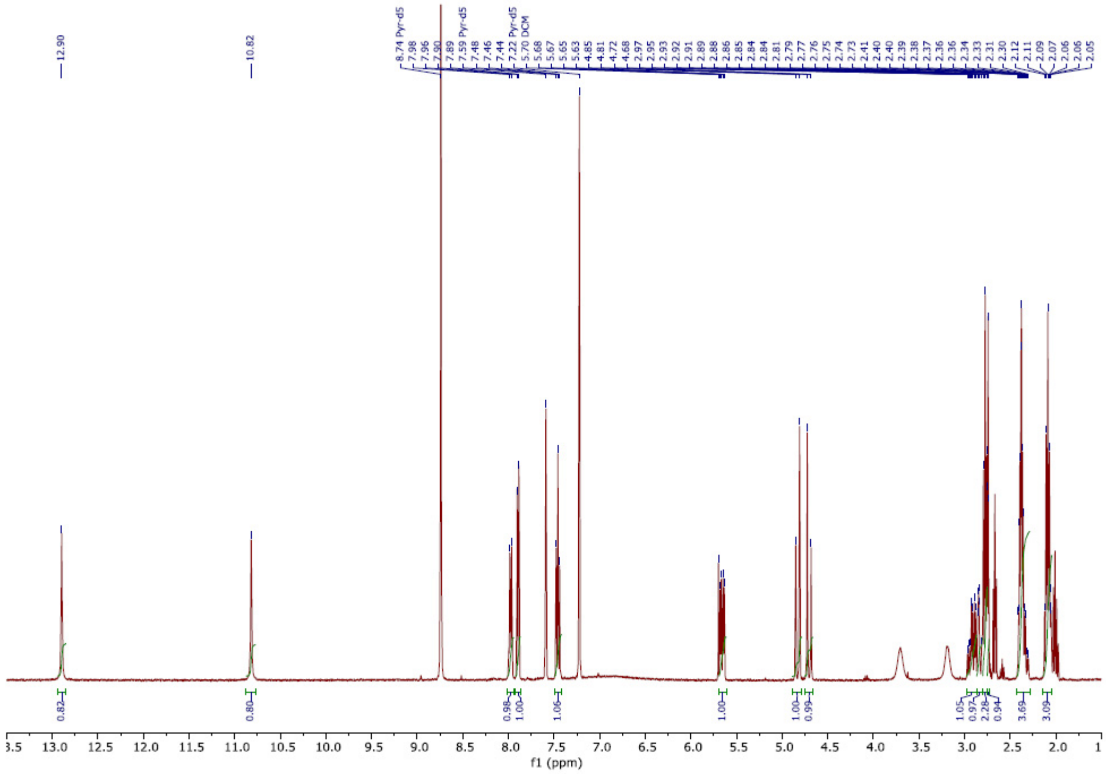

^{1}H NMR (400 MHz, pyridine-d5): δ (ppm) = 12.90 (s, 1H), 10.82 (s, 1H), 7.97 (d, *J* = 7.9 Hz, 1H), 7.89 (d, *J* = 7.6 Hz, 1H), 7.46 (t, *J* = 7.7 Hz, 1H), 5.66 (dd, *J* = 13.4, 5.1 Hz, 1H), 4.83 (d, *J* = 16.9 Hz, 1H), 4.70 (d, *J* = 16.9 Hz, 1H), 2.99 – 2.86 (m, 1H), 2.88 – 2.79 (m, 1H), 2.77 (t, *J* = 7.3 Hz, 2H), 2.74 (t, *J* = 2.6 Hz, 1H), 2.43 – 2.28 (m, 3H), 2.13 – 2.04 (p, *J* = 7.1 Hz, 2H + m, 1H).

Figure S8. ^{1}H-NMR spectra of **5**

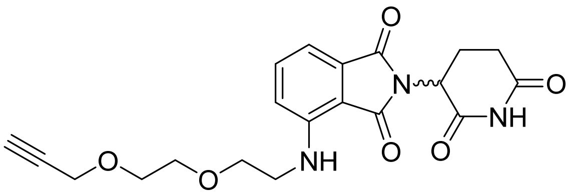

5

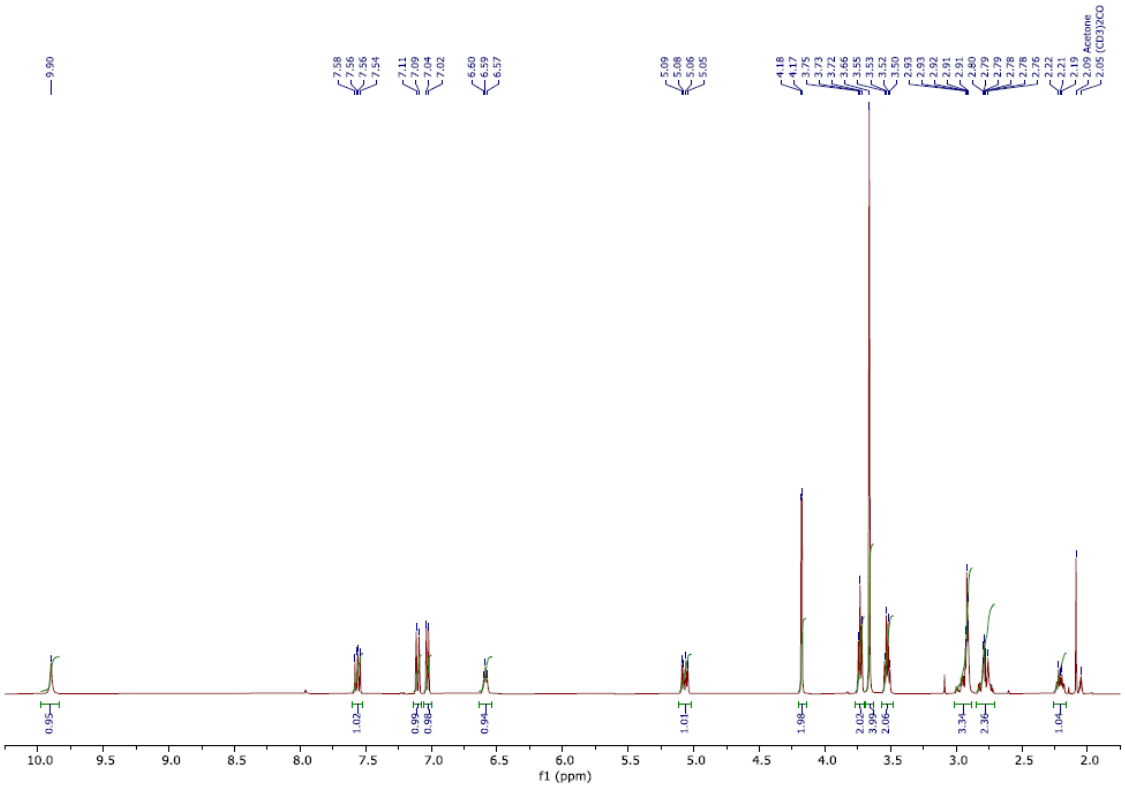

^{1}**H NMR** (400 MHz, acetone-d6): δ (ppm) = 9.90 (s, 1H), 7.56 (dd, *J* = 8.6, 7.0 Hz, 1H), 7.10 (d, *J* = 8.5 Hz, 1H), 7.03 (d, *J* = 7.1 Hz, 1H), 6.59 (t, *J* = 5.8 Hz, 1H), 5.07 (dd, *J* = 12.6, 5.4 Hz, 1H), 4.18 (d, *J* = 2.4 Hz, 2H), 3.73 (t, *J* = 5.4 Hz, 2H), 3.66 (s, 4H), 3.53 (q, *J* = 5.5 Hz, 2H), 3.02 – 2.86 (m, 3H), 2.85 – 2.70 (m, 2H), 2.26 – 2.16 (m, 1H).

Figure S9. ^{1}H-NMR spectra of **6**

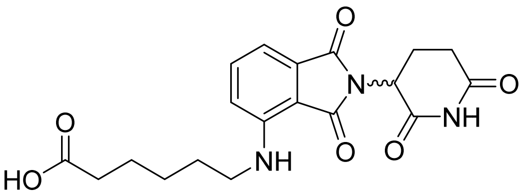

6

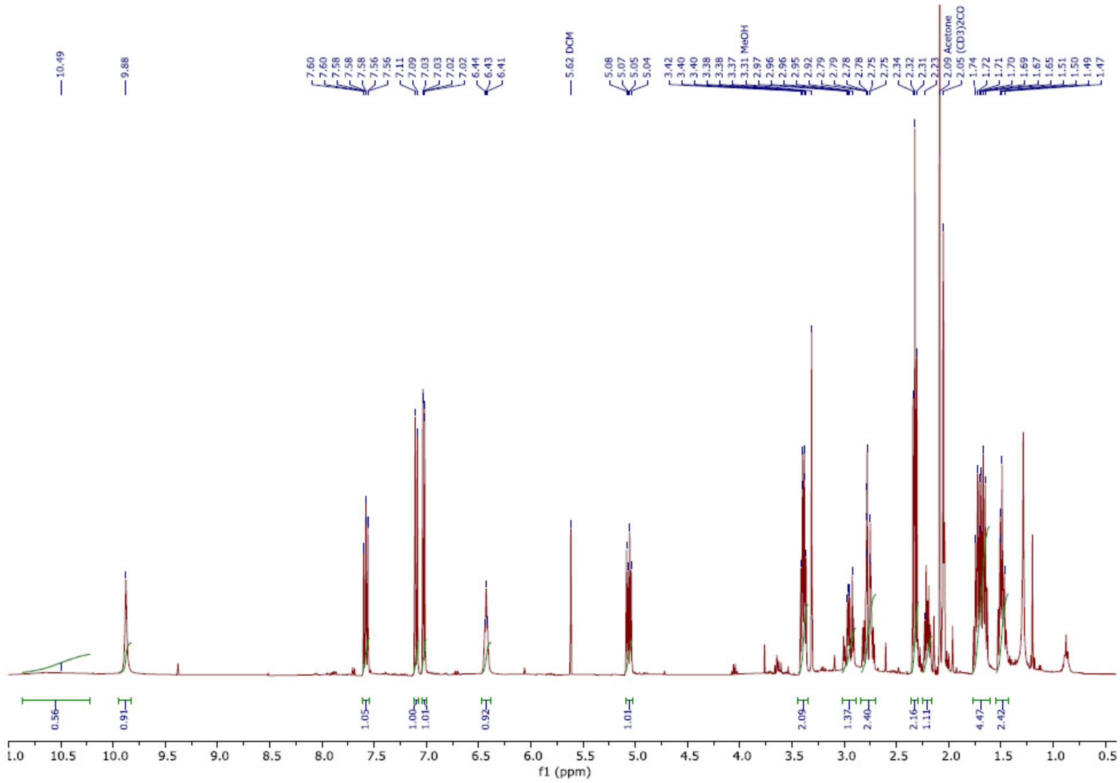

^{1}H NMR (400 MHz, acetone-d6): δ (ppm) = 10.49 (br, 1H), 9.88 (s, 1H), 7.58 (dd, *J* = 8.3, 7.3 Hz, 1H), 7.10 (d, *J* = 8.5 Hz, 1H), 7.02 (d, *J* = 7.1 Hz, 1H), 6.43 (t, *J* = 5.8 Hz, 1H), 5.06 (dd, *J* = 12.5, 5.4 Hz, 1H), 3.39 (td, *J* = 7.1, 5.8 Hz, 2H), 3.03 – 2.88 (m, 1H), 2.85 – 2.69 (m, 2H), 2.32 (t, *J* = 7.3 Hz, 2H), 2.24 – 2.15 (m, 1H), 1.78 – 1.59 (m, 4H), 1.55 – 1.42 (m, 2H).

Figure S10. ^{1}H and ^{13}C-NMR spectra of **7a**

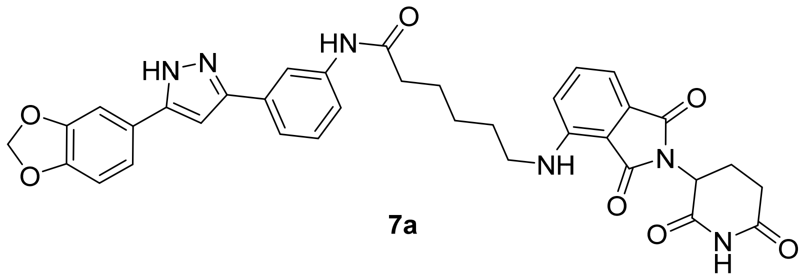

7a

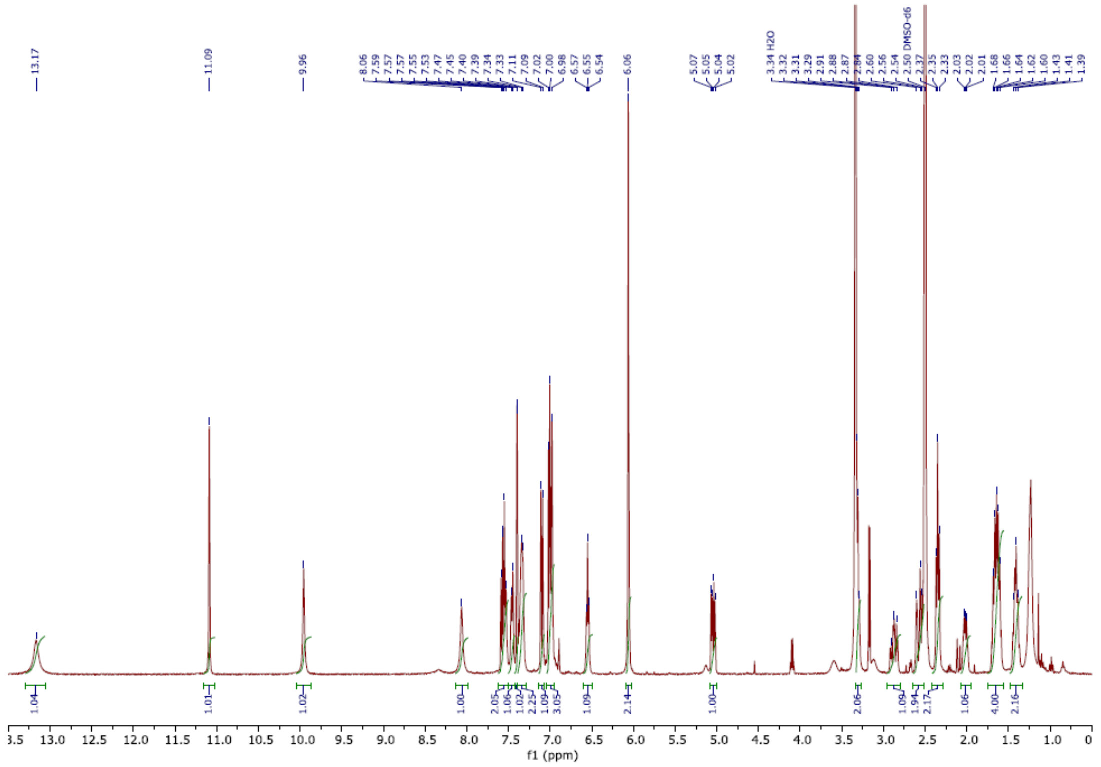

^{1}H NMR (400 MHz, DMSO-d6): δ (ppm) = 13.17 (s, 1H), 11.09 (s, 1H), 9.96 (s, 1H8.06 (s, 1H), 7.61 – 7.50 (m, 2H), 7.46 (d, *J* = 7.7 Hz, 1H), 7.40 (d, *J* = 1.7 Hz, 1H), 7.37 – 7.31 (m, 2H), 7.10 (d, *J* = 8.6 Hz, 1H), 7.04 – 6.95 (m, 3H), 6.55 (t, *J* = 6.1 Hz, 1H), 6.06 (s, 2H), 5.04 (dd, *J* = 12.9, 5.3 Hz, 1H), 3.32 – 3.27 (m, 2H), 2.94 – 2.81 (m, 1H), 2.62 – 2.48 (m, 2H), 2.35 (t, *J* = 7.3 Hz, 1H), 2.06 – 1.96 (m, 1H), 1.72 – 1.56 (m, 4H), 1.47 – 1.34 (m, 2H).

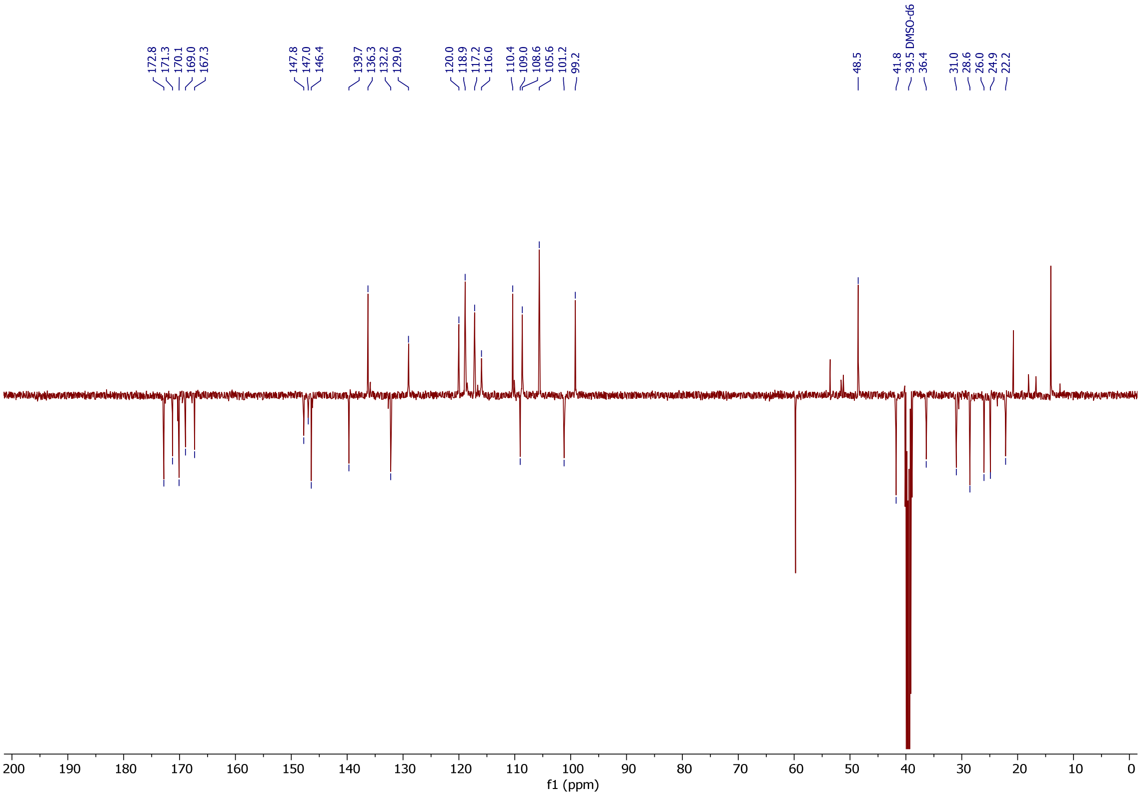

¹³C NMR (101 MHz, DMSO-d6)**:** δ (ppm) = 172.8, 171.3, 170.1, 169.0, 167.3, 147.8, 147.0, 146.4, 139.7, 136.3, 132.2, 129.0, 120.0, 118.9, 117.2, 116.0, 110.4, 109.0, 108.6, 105.6, 101.2, 99.2, 48.5, 41.8, 36.4, 31.0, 28.6, 26.0, 24.9, 22.2.

Figure S11. ^{1}H and ^{13}C-NMR spectra of **7b**

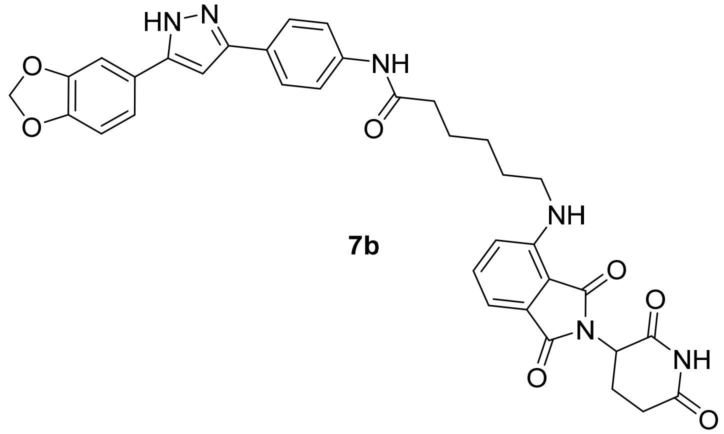

7b

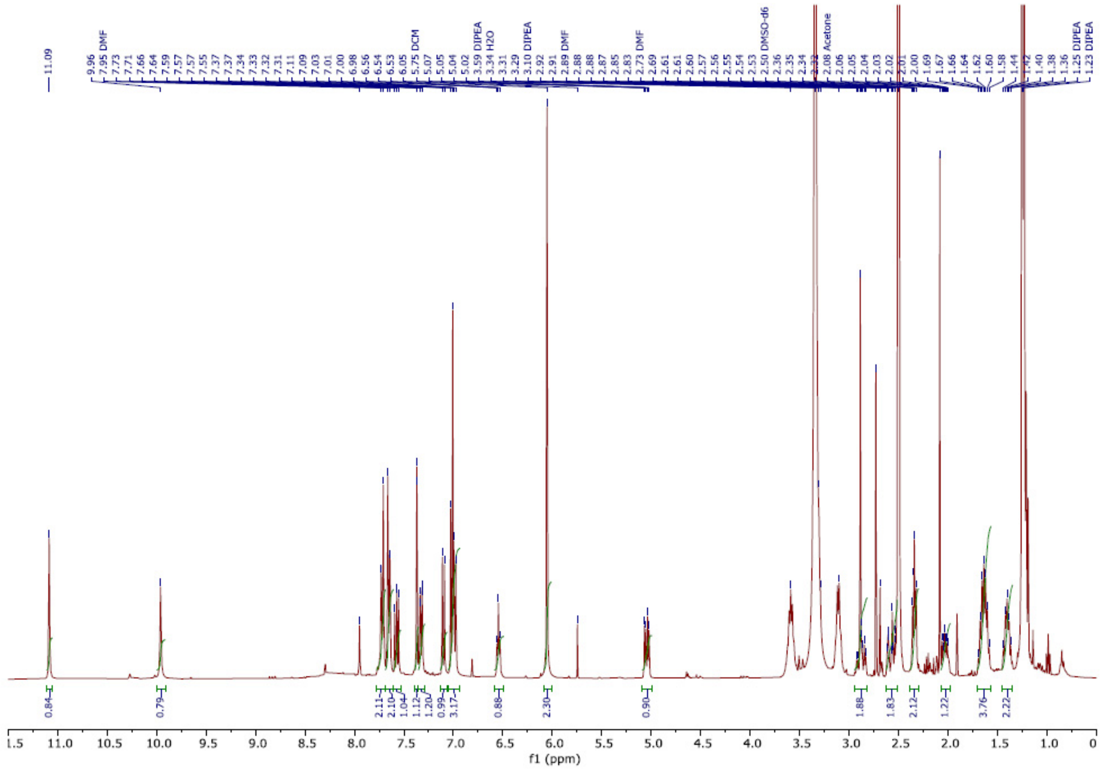

^{1}H NMR (400 MHz, DMSO-d6): δ (ppm) = 11.09 (s, 1H), 9.96 (s, 1H), 7.72 (d, J = 8.6 Hz, 2H), 7.65 (d, J = 8.7 Hz, 2H), 7.57 (dd, J = 8.6, 7.1 Hz, 1H), 7.37 (d, J = 1.7 Hz, 1H), 7.32 (dd, J = 8.0, 1.7 Hz, 1H), 7.10 (d, J = 8.6 Hz, 1H), 7.06 – 6.94 (m, 3H), 6.54 (t, J = 5.9 Hz, 1H), 6.05 (s, 2H), 5.05 (dd, J = 12.9, 5.4 Hz, 1H), 3.32 – 3.27 (m, 2H), 2.96 – 2.81 (m, 1H), 2.63 – 2.49 (m, 1H), 2.35 (t, J = 7.5 Hz, 2H), 2.07 – 1.97 (m, 1H), 1.72 – 1.56 (m, 4H), 1.46 – 1.33 (m, 1H).

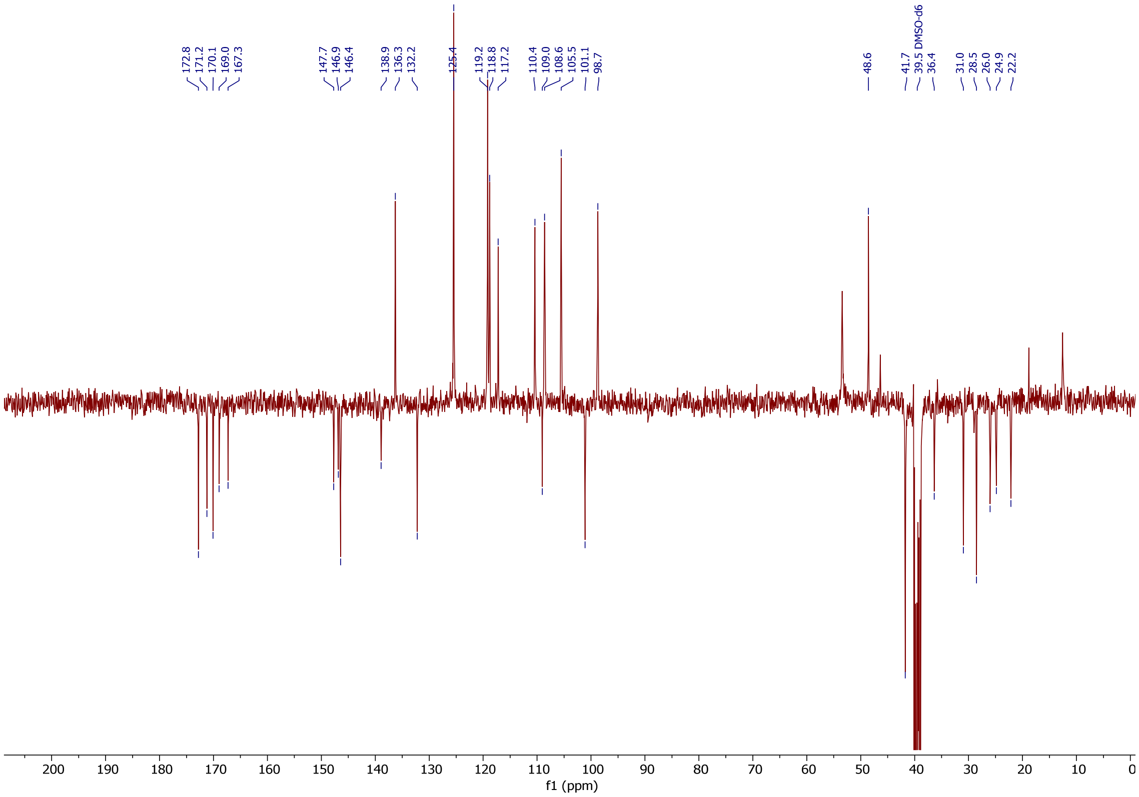

^{13}C NMR (101 MHz, DMSO-d6)**:** δ (ppm) = 172.8, 171.2, 170.1, 169.0, 167.3, 147.7, 146.9, 146.4, 138.9, 136.3, 132.2, 125.4, 119.2, 118.8, 117.2, 110.4, 109.0, 108.6, 105.5, 101.1, 98.7, 53.4, 48.6, 41.7, 36.4, 31.0, 28.5, 26.0, 24.9, 22.2, 18.8, 12.6.

Figure S12. ^{1}H and ^{13}C-NMR spectra of **8a**

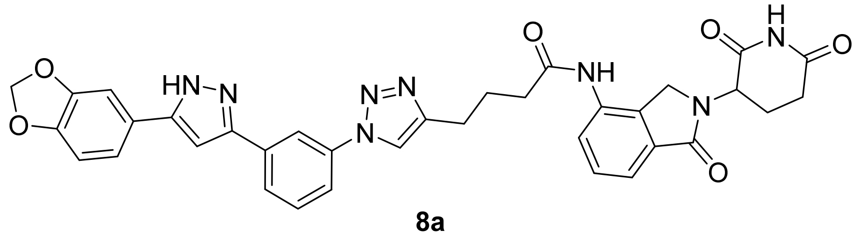

8a

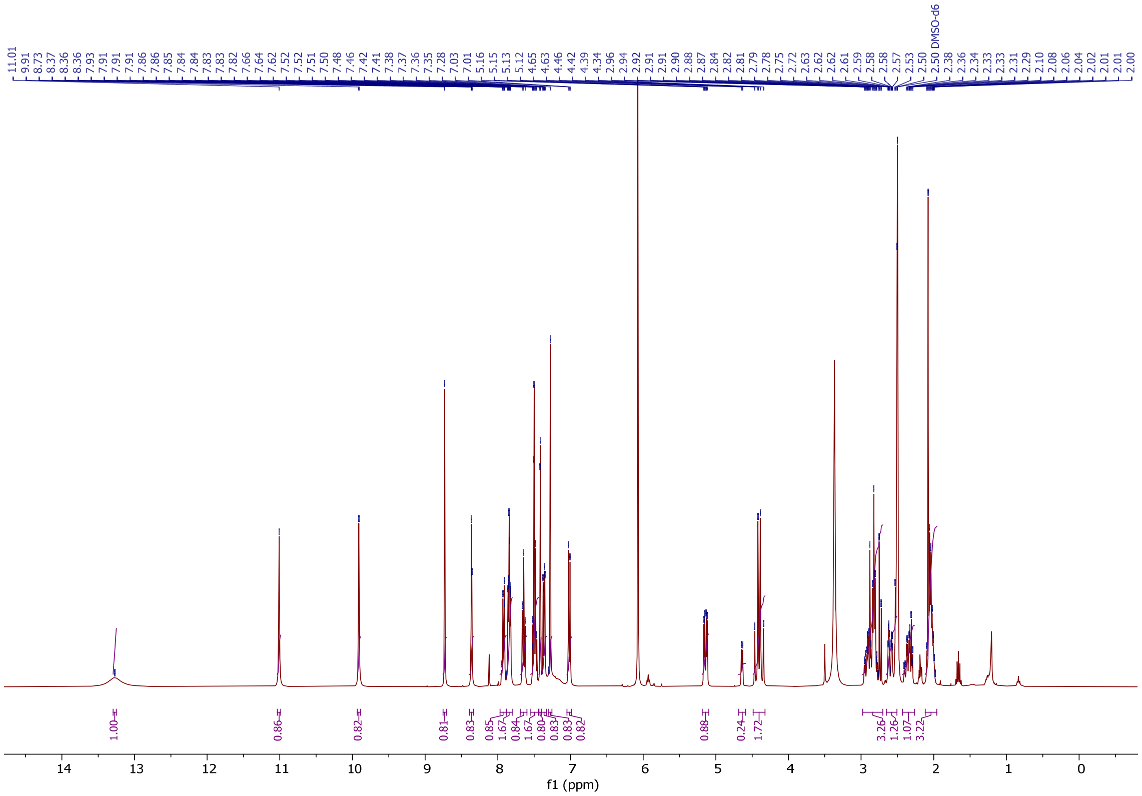

^{1}**H NMR** (400 MHz, DMSO-d6): δ (ppm) = 13.27 (s, 1H), 11.01 (s, 1H), 9.91 (s, 1H), 8.73 (s, 1H), 8.36 (t, J = 1.9 Hz, 1H), 7.97 – 7.88 (m, 1H), 7.84 (m, 2H), 7.64 (t, J = 7.9 Hz, 1H), 7.55 – 7.44 (m, 2H), 7.42 (d, J = 1.7 Hz, 1H), 7.36 (dd, J = 8.1, 1.7 Hz, 1H), 7.28 (s, 1H), 7.02 (d, J = 8.0 Hz, 1H), 5.14 (dd, J = 13.3, 5.1 Hz, 1H), 4.64 (d, J = 6.5 Hz, 1H), 4.49 – 4.32 (m, 2H), 2.98 – 2.70 (m, 4H), 2.65 – 2.51 (m, 1H), 2.43 – 2.27 (m, 1H), 2.12 – 1.96 (m, 4H).

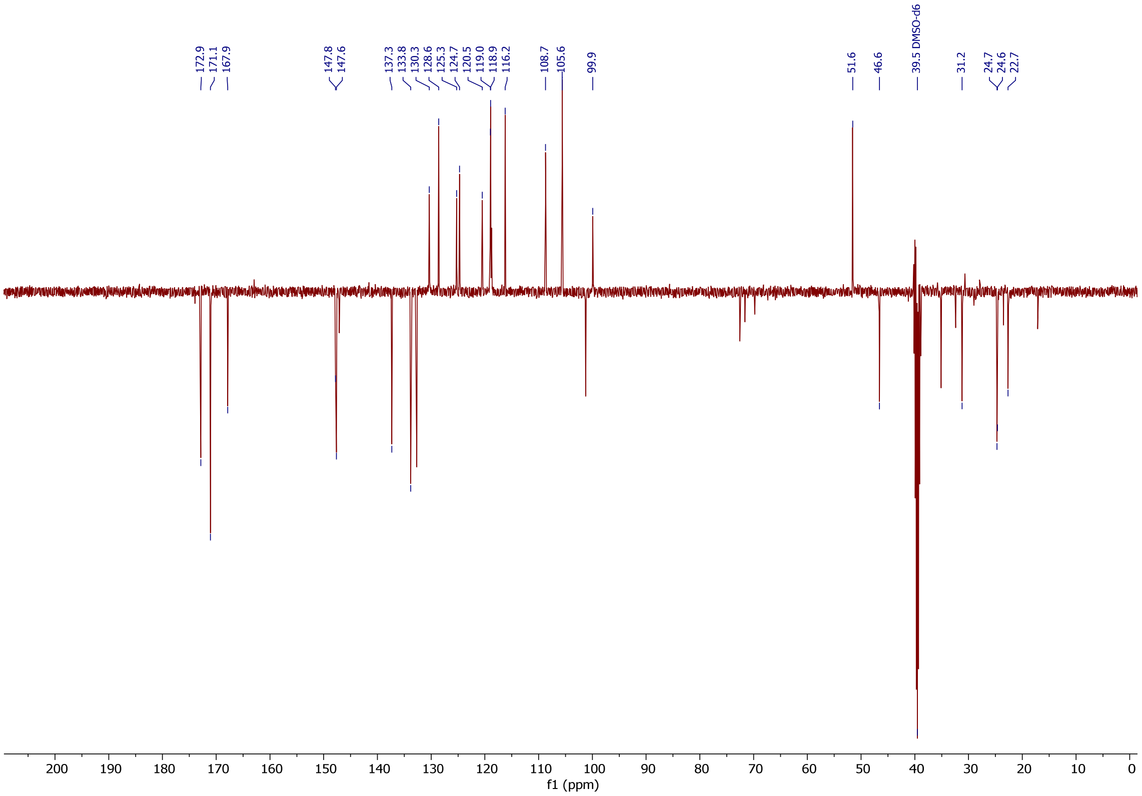

^{13}C NMR (101 MHz, DMSO-d6): δ (ppm) = 172.9, 171.1, 167.9, 147.8, 147.6, 137.3, 133.8, 130.3, 128.6, 125.3, 124.7, 120.5, 119.0, 118.9, 116.2, 108.7, 105.6, 99.9, 51.6, 46.6, 31.2, 24.7, 24.6, 22.7.

Figure S13. ^{1}H and ^{13}C-NMR spectra of **8b**

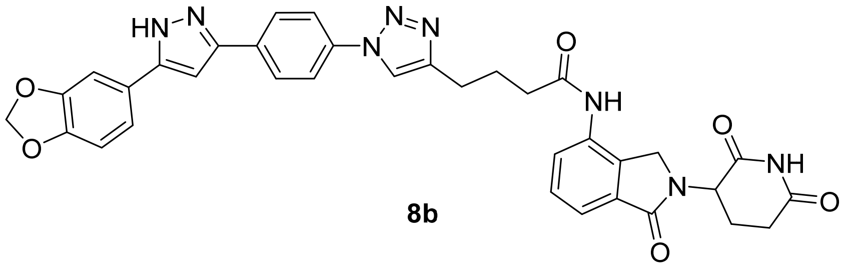

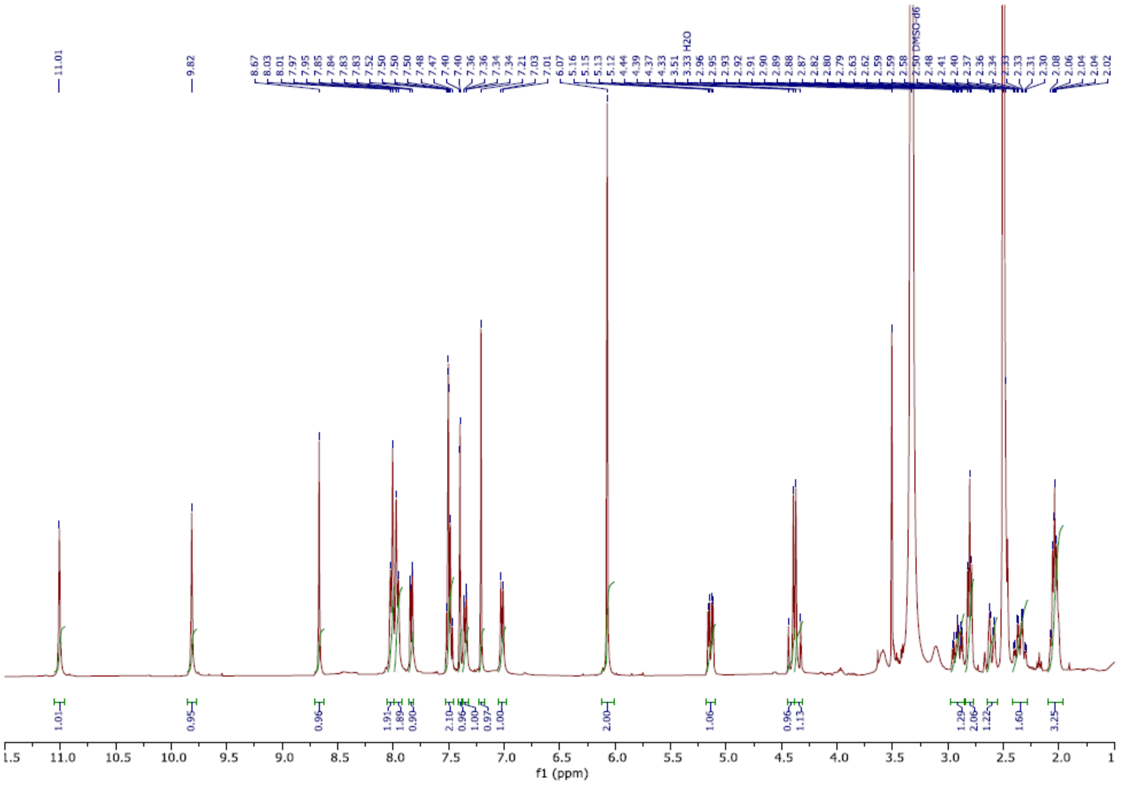

^{1}H NMR (400 MHz, DMSO-d6): δ (ppm) = 11.01 (s, 1H), 9.82 (s, 1H), 8.67 (s, 1H), 8.02 (d, *J* = 8.4 Hz, 2H), 7.96 (d, *J* = 8.5 Hz, 2H), 7.84 (dd, *J* = 6.9, 2.1 Hz, 1H), 7.55 – 7.42 (m, 2H), 7.40 (d, *J* = 1.7 Hz, 1H), 7.35 (dd, *J* = 8.2, 1.7 Hz, 1H), 7.21 (s, 1H), 7.02 (d, *J* = 8.0 Hz, 1H), 6.07 (s, 2H), 5.14 (dd, *J* = 13.3, 5.1 Hz, 1H), 4.42 (d, *J* = 17.6 Hz, 1H), 4.35 (d, *J* = 17.5 Hz, 1H), 2.92 (ddd, *J* = 17.2, 13.6, 5.4 Hz, 1H), 2.80 (t, *J* = 7.4 Hz, 2H), 2.66 – 2.55 (m, 1H), 2.52 – 2.44 (under solvent peak, 2H), 2.43 – 2.27 (m, 1H), 2.11 – 1.96 (m, 3H).

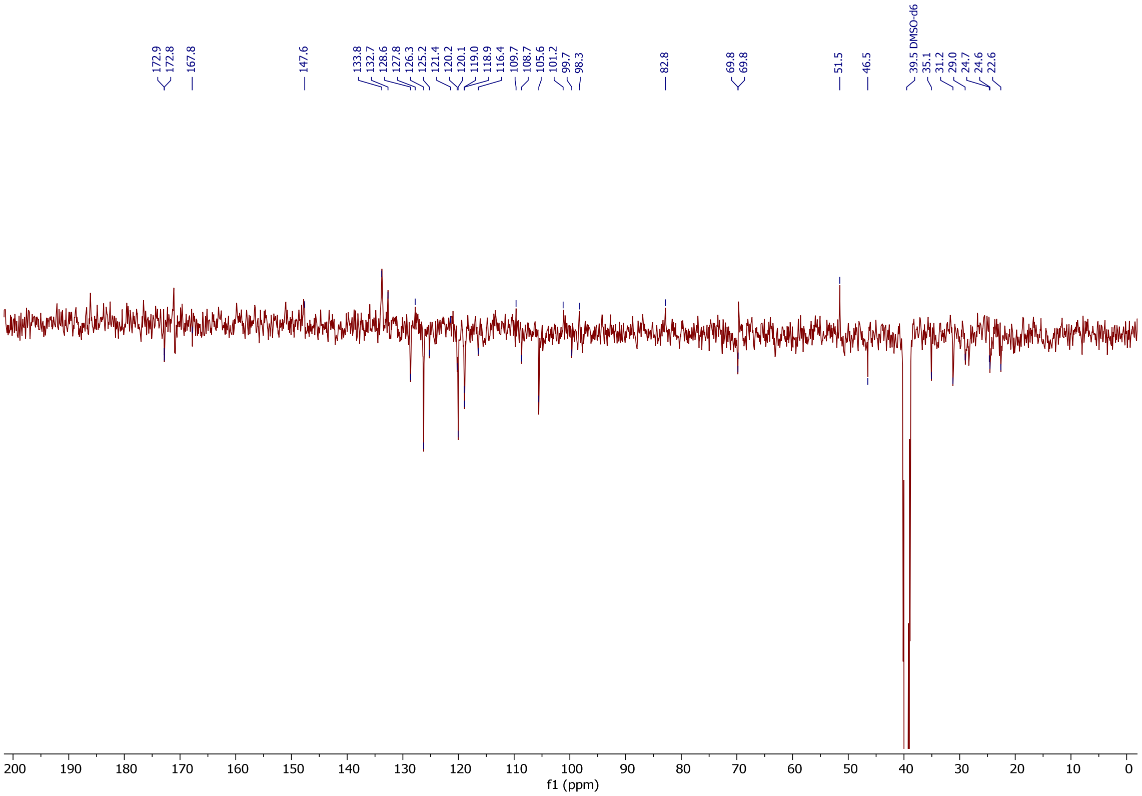

^{13}C NMR (101 MHz, DMSO-d6)**:** δ (ppm) = 172.9, 172.8, 167.8, 147.6, 133.8, 132.7, 128.6, 127.8, 126.3, 125.2, 121.4, 120.2, 120.1, 119.0, 118.9, 116.4, 109.7, 108.7, 105.6, 101.2, 99.7, 98.3, 82.8, 69.8, 69.8, 51.5, 46.5, 35.1, 31.2, 29.0, 24.7, 24.6, 22.6.

Figure S14. ^{1}H and ^{13}C-NMR spectra of **9a**

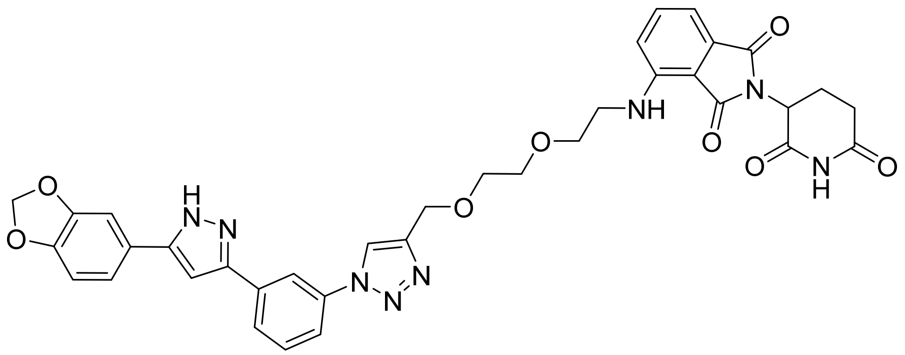

9a

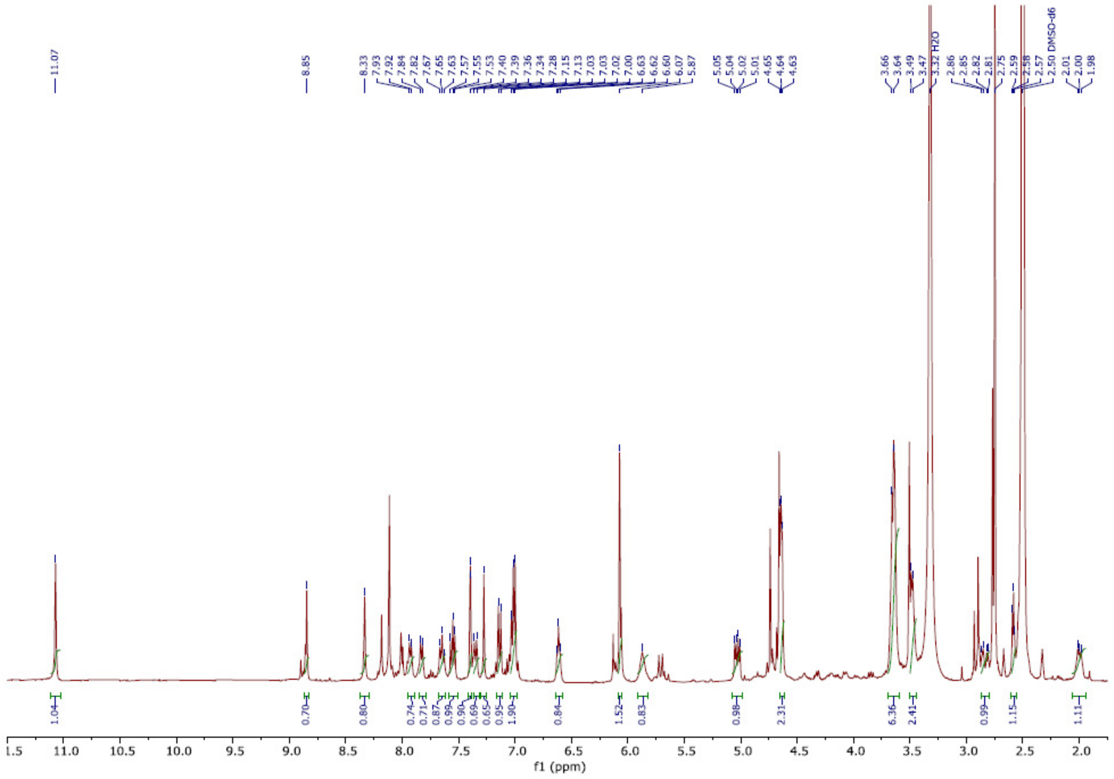

^{1}H NMR (400 MHz, DMSO-d6): δ (ppm) = 11.07 (s, 1H), 8.85 (s,1H), 8.33 (s, 1H), 7.93 (d, *J* = 7.3 Hz, 1H), 7.83 (d, *J* = 7.9 Hz, 1H), 7.65 (t, *J* = 7.9 Hz, 1H), 7.55 (t, *J* = 8.3 Hz, 1H), 7.40 (d, *J* = 1.3 Hz, 1H), 7.35 (d, *J* = 8.2 Hz, 1H), 7.28 (s, 1H), 7.14 (d, *J* = 8.6 Hz, 1H), 7.06 – 6.98 (m, 2H), 6.61 (t, *J* = 5.3 Hz, 2H), 6.07 (s, 2H), 5.03 (dd, *J* = 12.9, 5.4 Hz, 1H), 4.67 – 4.61 (m, 2H), 3.69 – 3.60 (m, 6H), 3.51 – 3.44 (m, 2H), 2.89 – 2.79 (m, 1H), 2.62 – 2.48 (m, 2H), 2.04 – 1.96 (m, 1H).

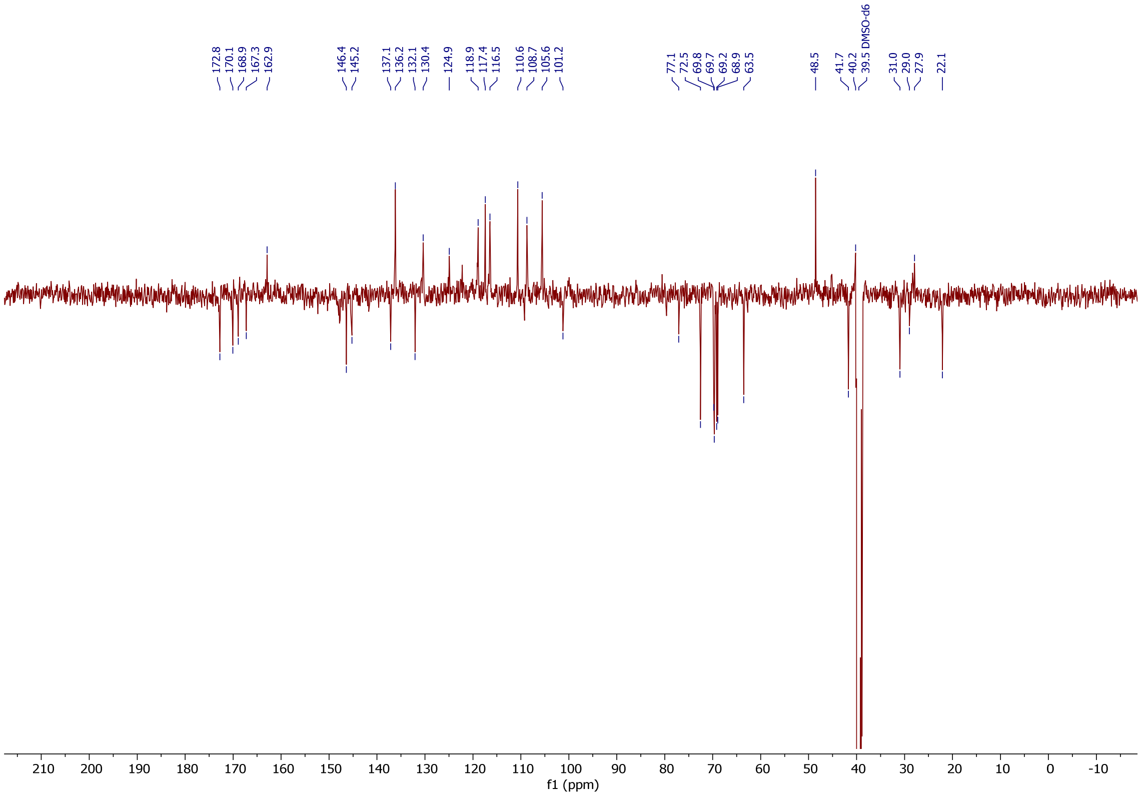

¹³C NMR (101 MHz, DMSO-d6)**:** δ (ppm) = 172.8, 170.1, 168.9, 167.3, 162.9, 146.4, 145.2, 137.1, 136.2, 132.1, 130.4, 124.9, 118.9, 117.4, 116.5, 110.6, 108.7, 105.6, 101.2, 77.1, 72.5, 69.8, 69.7, 69.2, 68.9, 63.5, 48.5, 41.7, 40.2, 31.0, 29.0, 27.9, 22.1.

Figure S15. ^{1}H and ^{13}C-NMR spectra of **9b**

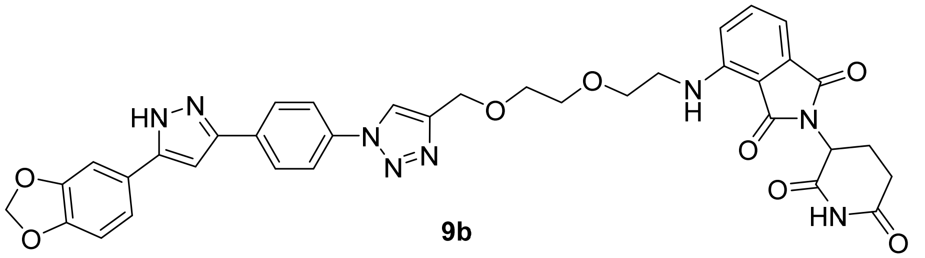

9b

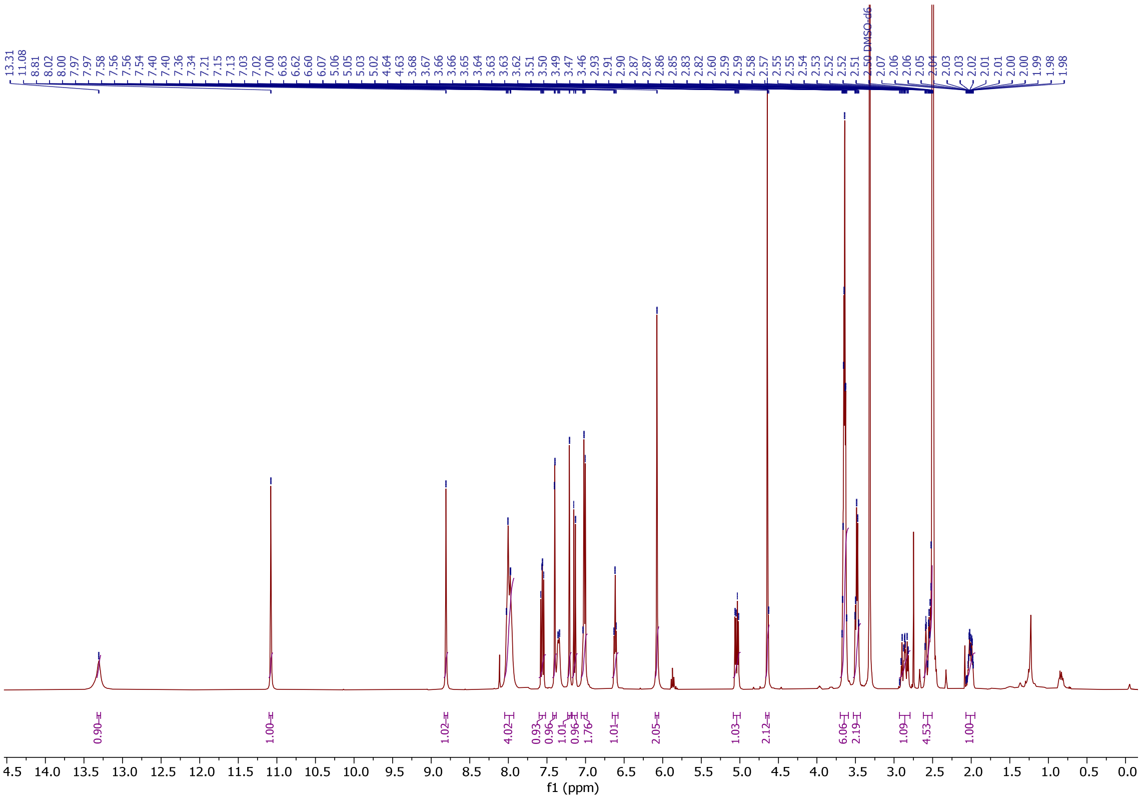

^{1}H NMR (400 MHz, DMSO-d6): δ (ppm) = 13.31 (s, 1H), 11.08 (s, 1H), 8.81 (s, 1H), 8.05 – 7.93 (m, 4H), 7.56 (dd, J = 8.6, 7.1 Hz, 1H), 7.40 (d, J = 1.7 Hz, 1H), 7.21 (s, 1H), 7.14 (d, J = 8.6 Hz, 1H), 7.01 (d, J = 7.0 Hz, 2H), 6.62 (t, J = 5.8 Hz, 1H), 6.07 (s, 2H), 5.04 (dd, J = 12.9, 5.4 Hz, 1H), 4.64 (s, 2H), 3.65 (m, 6H), 3.53 – 3.44 (m, 2H), 2.86 (m, 1H), 2.62 – 2.51 (m, 4H), 2.01 (m, 1H).

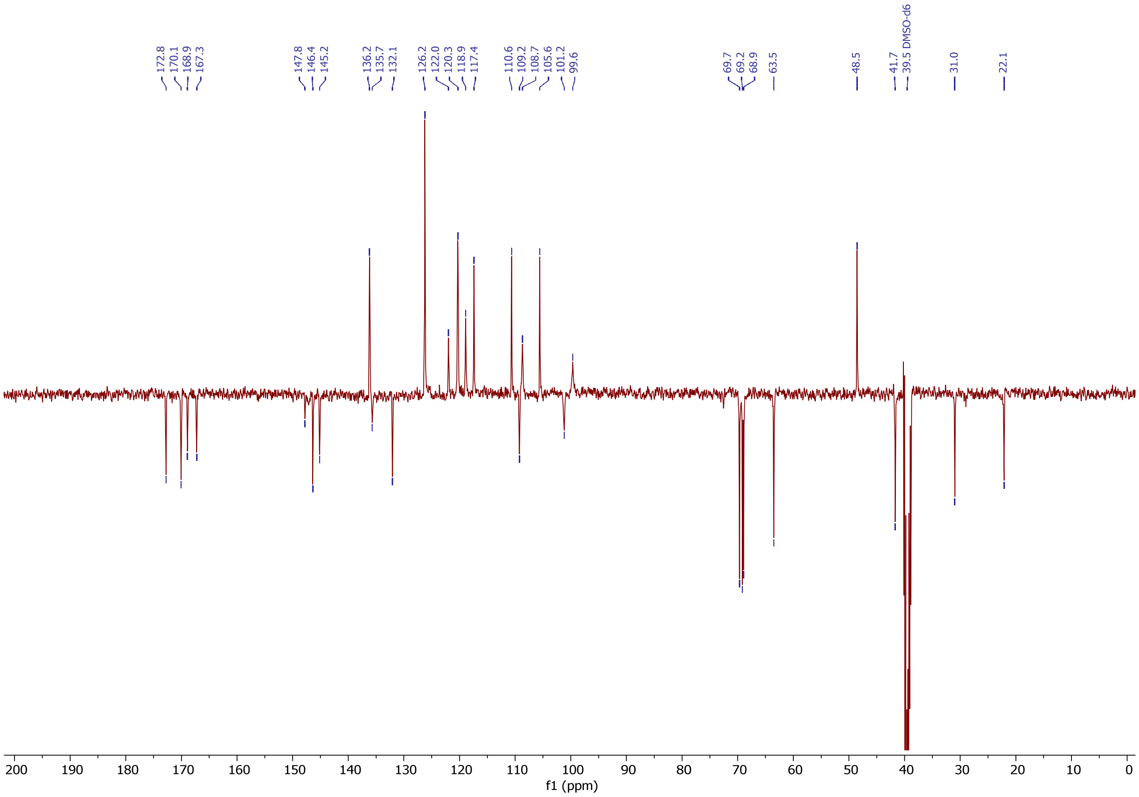

^{13}C NMR (101 MHz, DMSO): δ (ppm) = 172.8, 170.1, 168.9, 167.3, 147.8, 146.4, 145.2, 136.2, 135.7, 132.1, 126.2, 122.0, 120.3, 118.9, 117.4, 110.6, 109.2, 108.7, 105.6, 101.2, 99.6, 69.7, 69.2, 68.9, 63.5, 48.5, 41.7, 31.0, 22.1.

References

1. Joshi, Y. C.; Joshi, P.; Chauhan, S. S.; Nigam, S. Synthesis of novel pyrazole derivatives from dyaryl-1,3-diketones (Part II). *Heter. Commun*. **2004**, 10, 249-252.
